# A polygenic predictor of treatment-resistant depression using whole exome sequencing and genome-wide genotyping

**DOI:** 10.1038/s41398-020-0738-5

**Published:** 2020-02-03

**Authors:** Chiara Fabbri, Siegfried Kasper, Alexander Kautzky, Joseph Zohar, Daniel Souery, Stuart Montgomery, Diego Albani, Gianluigi Forloni, Panagiotis Ferentinos, Dan Rujescu, Julien Mendlewicz, Rudolf Uher, Cathryn M. Lewis, Alessandro Serretti

**Affiliations:** 1 Social, Genetic and Developmental Psychiatry Centre, Institute of Psychiatry, Psychology and Neuroscience, King’s College London, London, UK; 2 Department of Psychiatry and Psychotherapy, Medical University, Vienna, Austria; 3 Department of Psychiatry, Sheba Medical Center, Sackler School of Medicine, Tel Aviv University, Tel Hashomer, Israel; 4 Laboratoire de Psychologie Medicale, Universitè Libre de Bruxelles and Psy Pluriel, Centre Européen de Psychologie Medicale, Brussels, Belgium; 5 Imperial College School of Medicine, London, UK; 6 Laboratory of Biology of Neurodegenerative Disorders, Neuroscience Department, Istituto di Ricerche Farmacologiche Mario Negri IRCCS, Milan, Italy; 7 Department of Psychiatry, Athens University Medical School, Athens, Greece; 8 University Clinic for Psychiatry, Psychotherapy and Psychosomatic, Martin-Luther-University, Halle-Wittenberg, Germany; 9 Universite’ Libre de Bruxelles, Brussels, Belgium; 10 Department of Psychiatry, Dalhousie University, Halifax, NS Canada; 11 Department of Biomedical and NeuroMotor Sciences, University of Bologna, Bologna, Italy

**Keywords:** Predictive markers, Pharmacogenomics

## Abstract

Treatment-resistant depression (TRD) occurs in ~30% of patients with major depressive disorder (MDD) but the genetics of TRD was previously poorly investigated. Whole exome sequencing and genome-wide genotyping were available in 1209 MDD patients after quality control. Antidepressant response was compared to non-response to one treatment and non-response to two or more treatments (TRD). Differences in the risk of carrying damaging variants were tested. A score expressing the burden of variants in genes and pathways was calculated weighting each variant for its functional (Eigen) score and frequency. Gene-based and pathway-based scores were used to develop predictive models of TRD and non-response using gradient boosting in 70% of the sample (training) which were tested in the remaining 30% (testing), evaluating also the addition of clinical predictors. Independent replication was tested in STAR*D and GENDEP using exome array-based data. TRD and non-responders did not show higher risk to carry damaging variants compared to responders. Genes/pathways associated with TRD included those modulating cell survival and proliferation, neurodegeneration, and immune response. Genetic models showed significant prediction of TRD vs. response and they were improved by the addition of clinical predictors, but they were not significantly better than clinical predictors alone. Replication results were driven by clinical factors, except for a model developed in subjects treated with serotonergic antidepressants, which showed a clear improvement in prediction at the extremes of the genetic score distribution in STAR*D. These results suggested relevant biological mechanisms implicated in TRD and a new methodological approach to the prediction of TRD.

## Introduction

Major depressive disorder (MDD) is the second leading cause of disability in middle-aged adults on a global scale^1^. Despite the availability of a number of different pharmacological treatments, treatment-resistant depression (TRD) is estimated to occur in ~30% of patients^2^. TRD is usually defined as lack of response to at least two adequate treatments and it is associated with social and occupational impairment, suicidal thoughts, decline of physical health and increased health care utilization^3,4^. Annual costs for health care and lost productivity were estimated to be $5481 and $4048 higher, respectively, for a patient with TRD versus a patient with treatment-responsive depression^5^.

In the future, biomarkers associated with TRD risk may contribute to improve the clinical management of MDD by providing an estimate of TRD genetic risk at baseline, by guiding the prescription of personalized treatments and the development of new drugs. Genetic variants are ideal biomarkers to predict treatment response and TRD: a genetic basis to treatment response has been demonstrated and genotyping can be performed in easily accessible samples with reasonable cost and time^6^. The development of models able to predict the genetic risk of TRD at baseline would provide valuable information to personalize treatment prescription and hypothetically reduce the rate of TRD. Possible ways by which this could be achieved include: (1) identifying genetic predictors of non-response to specific antidepressant classes; (2) prescribing treatments with increased efficacy but limited availability because of costs constraints to patients having genetic risk for TRD. However, most existing pharmacogenomic studies were focused on measures of response to the last treatment without taking into account previous treatments, leaving the genetics of TRD largely unexplored^7^. Another issue was the investigation of common variants only, while the possible role of rare variants was overlooked, despite they were suggested as one of the factors contributing to missing heritability of common traits^8^. To the best of our knowledge, only a small pilot study (*n* = 10) performed whole exome sequencing to the study of treatment response in MDD (but not TRD) and found that the bone morphogenetic protein (*BMP5*) gene may be associated with the therapeutic outcome^9^.

The present study aimed to contribute in filling the existing gap in the knowledge of TRD genetics using whole exome sequencing and genome-wide genotyping to analyze the role of rare and common variants in the prediction of this phenotype and contribute to the development of predictive models potentially useful to personalize antidepressant prescription.

## Patients and methods

### Sample

The Group for the Study of Resistant Depression (GSRD) sample was recruited within a multicenter, cross-sectional study including adult in- and outpatients with MDD (DSM IV-TR criteria), as confirmed using the Mini International Neuropsychiatric Interview (MINI). Depressive symptom severity was assessed using the Montgomery and Åsberg Depression Rating Scale (MADRS) at study inclusion and at the onset of the current MDD episode. Information on previous and current antidepressant and other pharmacological treatments during the current MDD episode was collected as well as clinical-demographic characteristics. Antidepressant treatment was naturalistic according to best-clinical practice principles (Supplementary Table 1). The study protocol was approved by the local ethnic committees and the participant signed the written informed consent. Further details can be found elsewhere^10^.

### Phenotype, training, and testing samples

TRD was defined according to the most common definition of lack of response to at least two adequate antidepressant treatments during the current depressive episode^11^, while non-response was referred to one adequate treatment only. Adequate treatment was defined as an antidepressant treatment of minimum duration of 4 weeks at least at the minimum therapeutic dose according to drug labeling. Response was defined as a MADRS score <22 and a score decrease of at least 50% compared to the onset of the current MDD episode. Responders could have had not more than one failed antidepressant treatment during the current depressive episode. After quality control, the sample was split in a training (70%) and testing set (30%) which were balanced in terms of phenotypic distribution (TRD, non-response and response) using the partition function of groupdata2 R package, and they did not differ for gender, age, baseline depression severity, or centre of recruitment.

### Whole exome sequencing and genome-wide genotyping

Whole exome sequencing was performed using the Illumina HiSeq platform with 100 bp read length. Genome-wide genotyping was performed using the Illumina Infinium PsychArray 24 BeadChip (Illumina, Inc., San Diego) and these data were imputed as described in Supplementary Methods. Rare variants were extracted from exome sequence data and were defined as those having minor allele frequency (MAF) <1/√(2*n*), where *n* is the sample size^12^, which corresponded to 0.02 in GSRD.

Information about DNA extraction, quality control of exome sequence data and genome-wide data are reported as Supplementary Methods. We compared the concordance of genotypes of SNPs available in both exome sequence and array data, splitting them in genotyped and imputed and by MAF. These comparisons were also relevant to determine the putative reliability of rare imputed variants in the replication samples. Subjects with discrepancies between genome-wide and exome sequence data were excluded (non-major homozygote genotype concordance ≤90% for rare variants and ≤95% for common variants).

### Statistical analysis

#### Variant annotation and distribution of functional variants

We tested if predicted detrimental/damaging variants obtained through exome sequencing were differently distributed between TRD patients, non-responders, and responders. Variant annotation was performed using variant effect predictor (Vep) release 90, using the –pick flag that chooses one block of annotation per variant, based on an ordered set of criteria^13^. Annotations from SIFT, PolyPhen, and functional consequence scores from the sequence ontology (SO) project were used to estimate the relative pathogenicity of variants^14–16^. The use of scores which combine different variant annotations was also pursued and it is described in the next paragraph. The risk of carrying SIFT deleterious variants (scores < 0.05), PolyPhen damaging or probably damaging variants (scores > 0.45) and variants with SO functional score ≥ 0.90 and ≥0.70 in specific genes was compared across TRD patients, non-responders, and responders using regression models adjusted for three population principal components and center of recruitment. Bonferroni correction was applied to account for multiple testing (the number of included genes was between 14,353 and 18,600 depending from the considered annotation). Additional details are reported as Supplementary Methods.

#### Exome risk scores

These analyses aimed to estimate a weighted measure reflecting the burden of rare genetic variants exome-wide and in a gene-based and pathway-based way. Secondly, we combined these measures with analogous estimations for common variants.

For rare variants, a score was calculated for each individual as

$$\mathop {\sum}\limits_{i = 1}^n {v_{{\mathrm {all}}}{\times w_{\mathrm {s}}}{\times w_{\mathrm {f}}}}$$

where *n* is the number of genetic variants within the considered unit (whole exome, gene or pathway), *v*_all_ is the number of alternative alleles, *w*_s_ is the corresponding functional score and *w*_f_ is the frequency weight for that variant. In this way, the score is not dependent from the presence of individual variants which could not be observed in some of the tested samples. A similar approach was previously applied to the study of schizophrenia risk using exome sequence data^17^, but it was modified in this study by using different functional weighting (composite scores reflecting multiple annotations) and different frequency weighting (to allow the inclusion of rare but also common variants). Different sources for determining *w*_s_ were tested and compared (Eigen scores^18^, CADD scores^19^, and SO functional scores^15^, see Supplementary Methods). The frequency weight was determined using a beta distribution based on the frequency of the alternative allele alt_all (*w*_f_ = dbeta(alt_all,1,25), according to the previous literature^12^, see the corresponding curve in Supplementary Fig. 1). Rare variants were extracted from exome sequence data as those with MAF <1/√(2*n*), where *n* is the sample size^12^, which corresponded to 0.02 in GSRD. Common intragenic variants were extracted from genome-wide genotyping data and clumped based on their functional scores *w*_s_ and linkage disequilibrium (LD) using Plink v.1.9 (Supplementary Methods). A smoother beta distribution was used to weight these variants based on frequency (*w*_f_ = dbeta(alt_all,0.5,0.5)^12^, see curve in Supplementary Fig. 1).

The obtained scores were tested for different distribution among the phenotypic groups considering rare variants only and the sum of the scores for rare and common variants. These tests were performed using regression models adjusted for three population principal components and centre of recruitment.

#### Predictive modeling

Gene-based and pathway-based scores (adjusted for the described confounders, more details in Supplementary Methods) were entered into a predictor selection process in the training sample using a five-fold cross-validation repeated 100 times for pathways and 20 times for genes, 500 and 100 rounds in total, respectively. In each round, one-fifth of the training dataset was left out, and in the remaining four-fifths of the training dataset a correlation-adjusted T (CAT) score was estimated (i.e. a multivariate generalization of the standard univariate *T*-test statistic that takes the correlation among variables explicitly into account^20,21^) and the local false discovery rate (LFDR) (i.e. the probability of a variable to be non-informative with regard to phenotype prediction given its CAT score) for each potential predictor. We selected predictors that had a LFDR smaller than 0.8 in >50% of the rounds^22^. This process reduces dimensionality and select variables with higher probability of being informative, reducing the risk of overfitting. These predictors were used to develop predictive models in the training sample using a gradient boosting machine (GBM) algorithm with a five-fold cross-validation repeated 100 and 20 times when predictors were pathway and gene scores, respectively. Cross-validation in this phase was used to provide better estimates of predictor contribution and empirically estimate model parameters (number of trees and interaction depth; shrinkage was set to 0.1 and minimum number of observations in each terminal node was set to 10). GBM produces a prediction model in the form of an ensemble of weak prediction models based on decision trees and it was demonstrated to be a suitable algorithm to learn from weak predictors, when there is not a large amount of available data for training and predictors may interact among each other^23,24^. Models using gene-based scores as predictors included both rare and common variants, because the inclusion of rare variants only would have created scores very skewed towards zero which could not be realistically adjusted for confounders, while models using gene-set scores were tested for rare variants only and rare combined with common variants.

The performance of the developed models in predicting TRD or non-response in the testing sample was estimated using the area under the curve (AUC) of receiver operating characteristic (ROC) curves. Predictive models were developed in the whole training sample and in the subsamples treated with serotonergic antidepressants (5-HT ADs) and noradrenergic or noradrenergic–serotonergic antidepressants (NA ADs) according to the pharmacology domain reported in the NbN classification (Neuroscience-based Nomenclature)^25^. Different genetic profiles were indeed previously found for antidepressants belonging to these pharmacology domains^22^. Only the current treatment was considered and subjects treated with combinations of 5-HT ADs and NA ADs were not included in this analysis (Supplementary Table 1). The addition of a clinical risk score to the genetic predictors was evaluated. The clinical risk score was calculated as a weighted sum of the variables independently associated with TRD or non-response in the training sample in a regression model after Bonferroni correction (Supplementary Table 2). Each variable included in the clinical score was weighted for its effect size (*z* score) and divided by the number of variables available in each subject $({\mathop {\sum}\nolimits_{\mathrm {predictor} = 1}^n} {{\mathrm {predictor} \times z/n}})$ in order to avoid the exclusion of subjects with one or two missing values. We compared the ROC curves including genetic predictors with those including clinical or clinical-genetic predictors using the DeLong’s method.

The risk of TRD or non-response may increase particularly at the extremes of the genetic score distribution. Thus, we also tested the significant models including only subjects with a genetic score ≤30 or ≥70 percentiles; we used this threshold to balance the risk of instability of findings due to the limited sample size, particularly in the subsamples treated with specific drug classes. The total genetic score was calculated in each subject as a sum of the gene/pathway scores included in the model of interest, each of them weighted for its importance in the predictive model. This approach is a simplification, since it does not reflect the non-linearity of the developed models and possible interactions.

We did not perform multiple-testing correction for these analyses because: (1) these tests were correlated among each other and not independent (for example, patients in the tails of the genetic score are a subset); (2) we looked at the consistency of results of correlated analyses (i.e. we analyzed patients in the tails or added the clinical score for further testing models which showed non-random prediction in the basic test).

The following R cran packages were used for the described analyses: caret, nnet, sda, crossval, pROC.

### Replication

Replication of the significant predictive models was tested in STAR*D and GENDEP^26,27^, using the same approach described for creating gene-based and pathway-based risk scores (including rare and common variants according to the definition reported in the section “Exome risk scores”, more details are in Supplementary Methods). In replication samples we used a genetic score ≤20 or ≥80 percentiles to identify subjects with extreme genetic scores since the larger sample size. In both these samples genome-wide genotyping was available, including standard genome-wide arrays and an exome array (Illumina Infinium Exome-24 v1.0 BeadChip)^28^, but not exome sequence data. Further information on genotyping methods and quality control was previously reported^29^ and it is described also in the Supplementary Methods. Imputation was carried out using the Michigan imputation server and the Haplotype Reference Consortium (HRC, version r1.1 2016) as reference panel^30^. Different imputation quality thresholds were used to prune rare and common variants according to the previous literature (*R*^2^ > 0.30 and *R*^2^ > 0.60 for common and rare variants, respectively^31,32^). The comparability between the available rare variants in GENDEP/STAR*D and GSRD was tested in terms of number and functional annotation. Phenotypes were defined in a way comparable to the GSRD sample, only TRD and response were considered because of their univocal phenotypic definition (part of non-responders are expected to become TRD) and these analyses aimed to replicate significant results in GSRD (which were concentrated to the comparison TRD vs. response). Further details on phenotype definition are reported in Supplementary Methods (paragraph “Replication samples: STAR*D and GENDEP”).

### Power estimation

GSRD sample size after quality control (*n* = 1209) provides adequate power (≥0.80) in 865 out of 1000 simulations when testing a set of 45 simulated rare variants (MAF < 0.02) and 100 simulated common variants (which reflects the median number of variants in the analyzed genes), having effect sizes (*β*) randomly distributed between −0.25 and 0.25, at alpha = 2.69e–06 (Bonferroni corrected *p*-value for number of genes). R cran libraries KATSP, minqa and CompQuadForm were used for power estimation^33^.

## Results

The number of subjects available after quality control was 1209 (details on number of excluded subjects are in Supplementary Fig. 2). A comprehensive description of the clinical-demographic characteristics of the samples is reported in Supplementary Table 1, while a condensed overview is shown in Table 1. The number of included variants split by variant type and MAF is reported in Supplementary Table 3 (exome sequence data). Five subjects showed low concordance between genotypes available in both exome and genome-wide data and they were excluded from the analyses including both rare and common variants, since exome sequencing repeated on one of these subjects demonstrated genotype concordance >99% with the initial sequencing results. The comparison between sequenced rare variants and rare variants imputed from genome-wide data showed a mean concordance of 75% (SD = 5%) considering only non-major homozygote genotypes. The mean concordance considering the same comparison but for genotyped rare variants (array data) was 93% (SD = 2%) (Supplementary Fig. 3), suggesting that the use of rare variants obtained from an array may be feasible even though not optimal. From the genome-wide data, 476,319 intragenic common variants in low LD and 1180 subjects were included after quality control.

**Table 1:** Main clinical-demographic characteristics of the training sample (*n* = 847) and testing sample (*n* = 362).

Variable	Training sample (*n* = 847)	Testing sample (*n* = 362)
Age	51.44 ± 13.94	51.87 ± 14.16
Gender (F/M)	566/281	235/127
Phenotype of interest	TRD *n* = 353 Non-Responders *n* = 291 Responders = 203	TRD = 151 Non-responders *n* = 125 Responders = 86
Baseline MADRS score	34.56 ± 7.36	33.85 ± 7.69
Current MADRS score	24.73 ± 11.13	24.78 ± 11.60
Treatment	Serotonergic *n* = 421 Noradrenergic *n* = 271 Serotonergic–noradrenergic *n* = 128 Other *n* = 27	Serotonergic *n* = 192 Noradrenergic *n* = 93 Serotonergic–noradrenergic *n* = 59 Other *n* = 18

The baseline MADRS score is referred to the beginning of the current depressive episode. Mean ± standard deviation is reported for continuous variables and distribution for dichotomous ones. For a more comprehensive overview of patients’ characteristics and results of comparisons between the characteristics of the two subsamples see Supplementary Table 1.

*MADRS* Montgomery and Åsberg Depression Rating Scale; *TRD* treatment-resistant depression.

The variables included in the clinical risk score were suicidal risk, number of previous depressive episodes, chronic depression, and two MADRS factors (pessimism and interest-activity) (Supplementary Table 2).

### Distribution of damaging variants

**Fig. 1:**
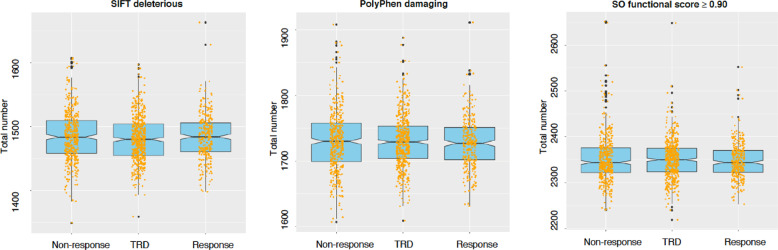
Representation of exome-wide distribution of variants with sequence ontology (SO) functional score ≥0.90, SIFT deleterious variants, PolyPhen damaging/probably damaging variants. The examined phenotypic groups (*x*-axis) were treatment-resistant depression (TRD), non-response, and response. The number of variants in each phenotypic group is reported on the *y*-axis.

Patients with TRD and non-responders did not show an increased risk to carry SIFT/PolyPhen damaging variants compared to responders or variants with SO functional score ≥0.90 or ≥0.70 (Supplementary Table 4 and Fig. 1). When considering individual genes (Supplementary Tables 5 and 6), we did not identify any difference among phenotypic groups after Bonferroni correction. The top gene was *WDR90* (WD Repeat Domain 90) which showed variants with SO functional score ≥0.90 in 21 patients with TRD but only in four non-responders and two responders (*p* = 3.44e–05).

### Exome-wide, gene, and pathway scores

The distribution of the of exome-wide scores for the three tested functional weights were substantially overlapping. Six patients were excluded from the subsequent analyses as they scored outside five standard deviations from the sample mean (Supplementary Fig. 4). Patients with TRD and non-responders did not show higher exome-wide scores compared to responders (*p* > 0.05 for all three tested functional weights). The correlations between gene scores calculated using the three tested functional weights were high (mean correlation coefficient between 0.89 and 0.95 with SD from 0.04 to 0.06 in pair-wise comparisons, Supplementary Fig. 5). In consideration of these high correlation coefficients, the demonstration that Eigen scores have better discriminatory ability using disease-associated and putatively benign variants from published studies compared to CADD scores^18^, and the lower functional precision of SO functional scores, only Eigen-based functional weighting was used in subsequent analysis.

Gene-based and pathway-based scores were not associated with phenotypic groups after Bonferroni correction (Supplementary Tables 7 and 8). The top genes were *NBN* and *ZNF418* (*p* = 4.34e–05 and 5.18e–05, respectively, whole sample, Supplementary Table 4) and the top pathways were protein interaction database (PID) CD40 pathway in the subsample treated with serotonergic drugs and GO (gene ontology) response to cocaine in the subsample-treated noradrenergic drugs (*p* = 5.28e–05 and 5.61e–05, respectively, Supplementary Table 8).

### Predictive modeling

**Fig. 2:**
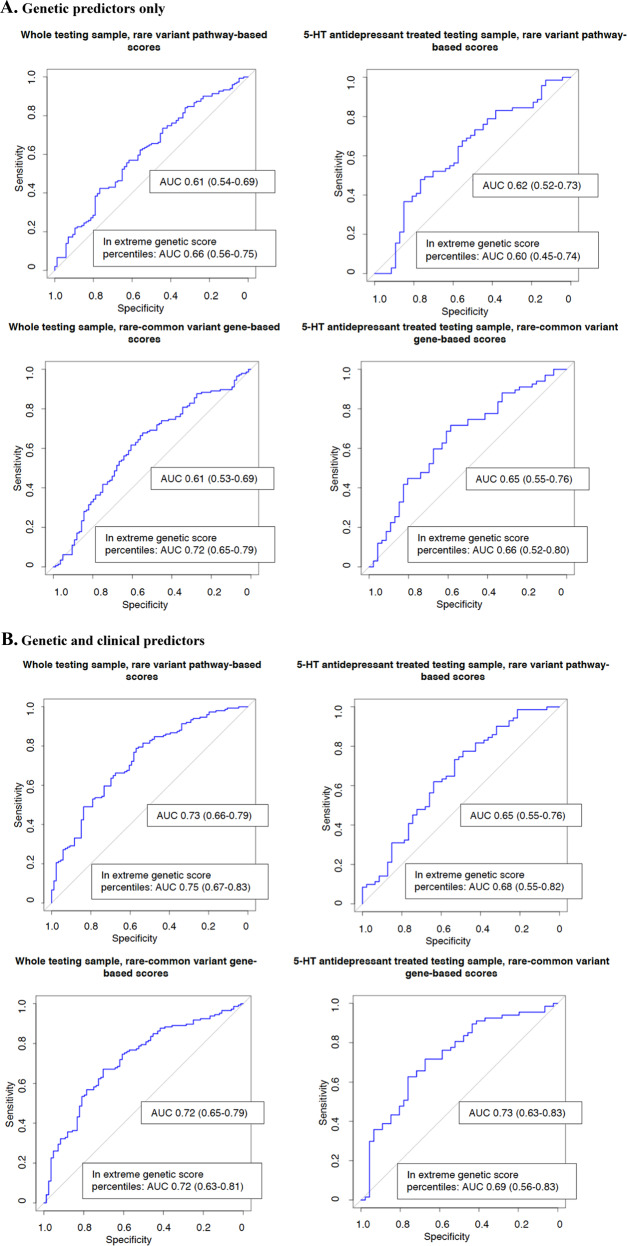
ROC curves of the non-random predictive models in GSRD testing sample and relative importance of the genetic predictors included in the models. When more than 20 predictors were included, only the first 20 are shown. 5-HT = serotonergic. The AUC values reached including only subjects with genetic scores ≤30 or ≥70 percentiles. **a** Genetic predictors only. **b** Genetic and clinical predictors.

Pathway-based models for TRD vs. response in the whole sample including only rare genetic variants showed non-random prediction in the testing sample (*n* = 237, AUC 0.61 [95% CI 0.54–0.69], Table 2 and Fig. 2) and in patients treated with 5-HT ADs (*n* = 272 and *n* = 118 in the training and testing samples, respectively, AUC 0.62 [95% CI 0.52–0.73], Table 2 and Fig. 2). The list of pathways used as predictors is in Supplementary Table 9. No significant prediction of TRD vs. response was observed in patients treated with NA ADs or when comparing non-responders vs. responders or TRD plus non-responders vs. responders (Supplementary Table 10). Prediction was improved by adding the clinical risk score to genetic predictors in both the whole sample and patients treated with 5-HT ADs (AUC 0.73 [0.66–0.79] and AUC 0.65 [0.55–0.76], respectively, Table 2 and Fig. 2), and this effect was more evident in subjects having extreme genetic scores for the included pathways (*n* = 142, AUC 0.75 [0.67–0.83] and *n* = 71, AUC 0.68 [0.55–0.82], respectively; Table 2 and Fig. 2). However, there was no significant difference between the AUC obtained using the clinical risk score and that of the models including genetic and clinical predictors (*p* = 0.89 and *p* = 0.68 for the whole testing sample and for 5-HT ADs, respectively). The clinical risk score showed similar or better AUC compared to the models including genetic predictors alone (*p* = 0.03 and *p* = 0.45 for the whole testing sample and for 5-HT ADs, respectively). A possible interpretation of this finding can be found in the observation that patients in the 5-HT ADs group had a lower clinical risk score compared to the others (*p* = 9.73e–09).

**Table 2:** characteristics of the models showing significant prediction in the testing sample for the phenotype TRD vs. response.

Subsample	Genetic predictors only	Genetic predictors + clinical risk score	Extreme genetic percentiles^a^, genetic predictors only	Extreme genetic percentiles^a^, genetic predictors + clinical score
(A) Pathway-based scores including rare variants				
Whole testing set	AUC 0.61 (0.54–0.69) Sens = 0.42; spec = 0.77; PPV = 0.76; NPV = 0.43	AUC 0.73 (0.66–0.79) Sens = 0.79; spec = 0.57; PPV = 0.76; NPV = 0.60	AUC 0.66 (0.56–0.75) Sens = 0.53; spec = 0.76; PPV = 0.78; NPV = 0.51	AUC 0.75 (0.67–0.83) Sens = 0.79; spec = 0.60; PPV = 0.76; NPV = 0.65
5-HT drugs only	AUC 0.62 (0.52–0.73) Sens = 0.48; spec = 0.77; PPV = 0.76; NPV = 0.49	AUC 0.65 (0.55–0.76) Sens = 0.73; spec = 0.53; PPV = 0.70; NPV = 0.57	AUC 0.60 (0.45–0.74)	AUC 0.68 (0.55–0.82) Sens = 0.75; spec = 0.61; PPV = 0.75; NPV = 0.61
(B) Gene-based scores including common and rare vairants				
Whole testing set	AUC 0.61 (0.53–0.69) Sens = 0.68; spec = 0.55; PPV = 0.72; NPV = 0.49	AUC 0.72 (0.65–0.79) Sens = 0.67; spec = 0.70; PPV = 0.80; NPV = 0.55	AUC 0.61 (0.51–0.71) Sens = 0.54; spec = 0.70; PPV = 0.72; NPV = 0.52	AUC = 0.72 (0.63–0.81) Sens = 0.57; spec = 0.82; PPV = 0.82; NPV = 0.57
5-HT drugs only	AUC 0.65 (0.55–0.76) Sens = 0.72; spec = 0.59; PPV = 0.72; NPV = 0.59	AUC 0.73 (0.63–0.83) Sens = 0.72; spec = 0.67; PPV = 0.76; NPV = 0.62	AUC 0.66 (0.52–0.80) Sens = 0.76; spec = 0.59; PPV = 0.74; NPV = 0.62	AUC 0.69(0.56–0.83) Sens = 0.70; spec = 0.67; PPV = 0.76; NPV = 0.60

The results of the other tested models are shown in Supplementary Table 9. AUC 95% confidence intervals are reported within parenthesis.

*Sens* sensitivity, *spec* specificity, *PPV* positive predictive value, *NPV* negative predictive value, *5-HT* serotonergic drugs.

^a^Including only subjects with a genetic score ≤30 percentile or ≥70 percentile see the section “Predictive modeling” of the main manuscript for more details.

Pathway-based models including rare and common genetic variants did not show predictive effect in the testing sample in almost all scenarios (Supplementary Table 10).

Gene-based models including rare and common variants predicted TRD vs. response in the whole testing sample and in subjects treated with 5-HT ADs (*n* = 230, AUC 0.61 [0.53–0.69]; *n* = 113, AUC 0.65 [0.55–0.76], respectively; Table 2). The lists of genes used as predictors is shown in Supplementary Table 9. The addition of the clinical risk score improved the prediction while the subgroups having scores in the extreme percentiles did not show different results (Table 2). There was no significant difference between the AUC of the model including only clinical predictors and that of the models including genetic and clinical predictors (*p* = 0.74 and *p* = 0.70 for the whole testing sample and for 5-HT ADs, respectively). The clinical risk score showed similar or better AUC compared to the models including genetic predictors alone (*p* = 0.02 and *p* = 0.50 for the whole testing sample and for 5-HT ADs, respectively).

Predictive models of non-response vs. response showed marginal significance in the whole sample (*n* = 211, AUC 0.59 [0.51–0.67]) but better values in the sample treated with 5-HT ADs (*n* = 121, AUC 0.64 [0.53–0.74]; Supplementary Table 10). However, given that models including non-responders were significant in a smaller number of scenarios compared to those focused on TRD, we did not further investigate them in the replication samples.

### Replication in STAR*D and GENDEP

Despite the availability of genotypes from an exome array, a low covering of coding regions was obtained compared to exome sequence data, limiting the comparability of these data with those available in GSRD (Supplementary Fig. 7). In GENDEP, *LCE1B* gene was not covered and we had to re-train the corresponding predictive model (gene scores in patients treated with 5-HT ADs) without this gene, with no major change in predictive performance in the GSRD testing sample (not shown). The number of included subjects and their main clinical-demographic characteristics are reported in Supplementary Table 11.

None of the models including only genetic variables predicted TRD, apart from the rare variant pathway-based model developed in patients treated with 5-HT ADs. In STAR*D, this genetic model showed significant prediction of TRD risk in subjects with scores ≤10 or ≥90 percentiles (we looked at more extreme percentiles because of the larger sample size; *n* = 134, AUC 0.73, 95% CI 0.61–0.86, Table 3). The AUC of this model was not different from that of the model including clinical and genetic variables (*p* = 0.63), but it was better compared to the model including clinical variables only (AUC of the clinical predictor: 0.55 [0.49–0.62], *p* = 0.01). The other models showing replication (all including genetic and clinical predictors) are reported in Table 3; the ROC AUC of these models were not significantly different from those of the models based on the clinical risk score (all *p* > 0.05). An overview of all replication results is provided in Supplementary Table 12.

**Table 3:** best predictive models of treatment-resistant depression (TRD) vs. response in the replication samples.

Sample	Genetic predictors	Genetic predictors AUC (95% CI)	Clinical and genetic predictors AUC (95% CI)
GENDEP, whole sample (*n* = 321)	Pathways, rare variants	0.54 (0.47–0.60)	0.60 (0.54–0.65)
GENDEP, 5-HT antidepressants (*n* = 188)	Genes, rare and common variants	0.58 (0.49–0.68)	0.62 (0.53–0.72)
STAR*D, whole sample (*n* = 807)	Genes, rare and common variants	0.51 (0.46–0.55)	0.55 (0.51–0.59)
STAR*D, 5-HT antidepressants, ≤20 or ≥80 percentiles (*n* = 266)	Pathways, rare variants	0.59 (0.48–0.69)	0.61 (0.51–0.71)
STAR*D, 5-HT antidepressants, ≤10 or ≥90 percentiles (*n* = 134)	Pathways, rare variants	0.73 (0.61–0.86)	0.72 (0.58–0.86)

For a detailed overview of all results in the replication samples see Supplementary Table 12. In STAR*D, more extreme percentiles of the genetic predictors were considered compared to other samples because of the larger sample size (for details see the section “Replication in STAR*D and GENDEP”). 5-HT antidepressants = serotonergic antidepressants, *Cl* confidence intervals.

## Discussion

This study found no overall difference in the distribution of functional and deleterious/damaging variants between TRD patients, non-responders, and responders within the whole exome or within individual genes. The closest gene to the significance threshold was WD Repeat Domain 90 (*WDR90*), which product function is still poorly known but it is thought to participate in microtubule organization within the presynaptic axon terminal^34^. The tested risk scores were not associated with TRD at gene or gene set level, with *NBN* (nibrin) and *ZNF418* (Zinc Finger Protein 418) genes, PID CD40 and GO response to cocaine pathways as top results. *NBN* is thought to be involved in DNA double-strand break repair, DNA damage-induced checkpoint activation and telomere integrity^35^. It may be involved in neurodegenerative disorders^36^. Variants in the *ZNF418* region had a non-significant trend of association with MDD in a previous Psychiatric Genetic Consortium (PGC) mega-analysis^37^ and in an exome sequence study^38^. The PID CD40 gene set includes 31 genes, it is involved in the modulation of inflammation and CD40 ligand has been previously associated with MDD^39^.

The lack of strong signals coming from individual genes or pathways was expected as it is in line with a previous genome-wide association study of copy number variants (CNVs) that reported no significant enrichment of CNVs in TRD^40^. Thus, it is reasonable to hypothesize that if genetics contributes to TRD, multiple genes/pathways must be involved with complex interactions. This mirrors the highly polygenic liability to MDD that is emerging from other studies^41^. On the basis of this hypothesis, we applied predictive modeling to assess TRD risk using gene- and pathway genetic as well as clinical scores as predictors. Predictive modeling combining genetic and clinical predictors has been used by only two previous studies to predict antidepressant response to the best of our knowledge^22,42^, both these studies used SNPs from genome-wide genotyping as genetic predictors. In contrast to the present study, they did not perform any independent replication and the second study did not distinguish between training and testing sets^42^.

The present study applied an innovative approach which combined gene and pathway polymorphisms in genetic scores weighted by their functional relevance, using exome sequence and genome-wide data. The predictive models comparing TRD vs. response showed significant prediction in a higher number of scenarios compared to models including non-responders, confirming the biological relevance of TRD as a distinct phenotype. In this regard, it should be noted that non-responders are a more heterogeneous group than TRD patients, because part of them is expected to develop TRD. In the GSRD testing sample, both gene-based and pathway-based models showed significant prediction of TRD vs. response (Table 2). The genes/pathways included in these models (Supplementary Table 9) are mostly involved in cell survival, cell growth and replication, cell migration, neurodegenerative processes, neuroplasticity, immune system, hormonal regulation (sex and thyroid hormones) and second messenger cascades. Predictive performance was often improved by adding clinical risk factors and in the extreme percentiles of the score distribution. However, none of the genetic or genetic-clinical models showed a significantly better ROC AUC compared to the model including the clinical risk score only. We hypothesized two possible scenarios which could make the genetic predictors useful: (1) in patients with no clinical risk factors; (2) in patients having genetic scores at the extremes of the distribution. We preliminary tested the first hypothesis in GSRD whole testing sample: the pathway-based model showed AUC of 0.67 (0.54–0.81) in patients with no clinical risk factors (*n* = 64) vs. AUC = 0.61 (0.54–0.69) in the whole testing sample. The number of patients was limited (for this reason we did not explore this hypothesis in other subsamples), but the result supports the hypothesis that our genetic predictors perform slightly worse in patients with clinical risk factors, presumably because they are largely independent from them (i.e. genetic factors are not able to predict TRD cases caused by clinical variables having a distinct genetic or environmental basis). In line with this, there was no correlation between the cumulative genetic score (for any model) and the clinical risk score and genetic models were not able to predict TRD classification according to the clinical risk score. We hypothesized that the high impact of clinical risk factors in GSRD (most patients were complex cases of MDD, recruited in tertiary health care centres) may have led to a relative down-weighting of genetic predictors in the clinical-genetic models (Supplementary Fig. 6), explaining the fact that they did not show better performance in predicting TRD compared to the models including only the clinical risk score. We could not explore the contribution of the individual risk variables included in the risk score, because we used a cumulative score aimed to avoid the exclusion of subjects with partially missing data.

The fact that genetic models developed in patients treated with 5-HT AD had better AUC point estimates (Table 2) may be explained by the fact that these patients had significantly lower clinical risk factors compared to the others (*p* = 9.73e–09), since treatment prescription was naturalistic in GSRD. This means that the different gene/pathways selected in the whole sample compared to those selected in patients treated with 5-HT ADs may reflect their different clinical characteristics rather than differences due to distinctive biological mechanisms implicated in response to different drug classes. None of the analyses performed in the group treated with NA ADs was significant, a probable consequence of the small size of this group. In this regard, we also underline that polypharmacy was frequent in this sample, including combination and augmentation strategies^10^, thus our classification according to the antidepressants pharmacology represented a simplified approach.

The second scenario in which genetic predictors may be more relevant is in subjects with genetic risk scores at the extremes of the distribution. This case is exemplified by the clear improvement of prediction in subjects with genetic scores ≤10 or ≥90 percentiles in STAR*D (Table 3), the largest available sample in our study, which allowed to test more extreme percentiles compared to GSRD and GENDEP (at least the top 5% of the distribution was suggested to be meaningful for increased risk when using polygenic risk scores^43^, but we had no power for this). The corresponding model was the only one showing replication of genetic predictors only and superiority over the clinical risk score, while prediction in other models showing replication in STAR*D or GENDEP was driven by the clinical score. Unfortunately, the genetic data available in the replication samples were poorly comparable with those available in GSRD (only arrays, with low coverage of coding regions) and there were also clinical differences between STAR*D, GENDEP, and GSRD. For example, patients in STAR*D had very long depressive episodes of relatively mild severity, while in GENDEP there were no patients with chronic MDD according to the standard definition (≥2 years) and they had on average a lower number of previous episodes (Supplementary Table 11). Unlike the other samples, MADRS was not available in STAR*D and equivalent scores were calculated using the QIDS-C16 scale (Supplementary Methods). The definition of the phenotype was performed slightly differently in each sample, because of the differences in study design.

Bearing in mind the discussed limitations, our results contributed to clarify the genetic factors involved in TRD and it was the first study to assess the contribution of rare genetic variants through whole exome sequencing, if we exclude a very small pilot study performed on 10 subjects^9^. No individual gene or pathway probably plays a major role in TRD, thus models including multiple genes/pathways and able to account for their interactions are probably the best strategy. Theoretically, pathway-based models are more suitable to take into account the complex genetic component of antidepressant response compared to gene-based models and they are expected to be more replicable, as confirmed by our top replication results. Our study represents a new approach to the prediction of treatment resistance in MDD and future improvements in larger samples may lead to clinical applications, at least in patients with extreme genetic scores or those with no clinical risk factors. In patients having genetic risk for TRD, treatment strategies with demonstrated higher efficacy (e.g. pharmacotherapy combined with psychotherapy^44^) but limited availability for cost constraints could be implemented as first line treatment, when these patients first seek treatment and there are still no clinical signs of severe MDD and no clinical risk factors for TRD, reducing the proportion of patients at risk who progresses towards resistance.

## Supplementary information

Supplementary Materials.

## Data Availability

Genetic and clinical data from STAR*D study can be obtained by submitting an application at: https://www.nimhgenetics.org. Genetic and clinical data from GENDEP can be obtained by submitting an inquiry to cathryn.lewis@kcl.ac.uk. Genetic and clinical data from GSRD is available to analysts of the Psychiatric Genomics Consortium (https://www.med.unc.edu/pgc/), for enquires contact the corresponding author (alessandro.serretti@unibo.it).
