# Supplementary material for: A polygenic predictor of treatment-resistant depression using whole exome sequencing and genome-wide genotyping
Source: Transl Psychiatry. 2020 Feb 3;10:50. doi: 10.1038/s41398-020-0738-5 (PMC7026437; doi:10.1038/s41398-020-0738-5)
Supplement: Supplementary file 1 — Supplementary Materials. [file 41398_2020_738_MOESM1_ESM.pdf]

Supplementary Materials

A polygenic predictor of treatment-resistant depression using whole exome sequencing and genome-wide genotyping

Chiara Fabbri [1], Siegfried Kasper [2], Alexander Kautzky [2], Joseph Zohar [3], Daniel Souery [4], Stuart Montgomery [5], Diego Albani [6], Gianluigi Forloni [6], Panagiotis Ferentinos [7], Dan Rujescu [8], Julien Mendlewicz [9], Rudolf Uher [10], Cathryn M. Lewis [1], Alessandro Serretti [11]

1: Social, Genetic and Developmental Psychiatry Centre, Institute of Psychiatry, Psychology and Neuroscience, King's College London, United Kingdom

2: Department of Psychiatry and Psychotherapy, Medical University Vienna, Austria

3: Department of Psychiatry, Sheba Medical Center, Sackler School of Medicine, Tel Aviv University, Tel Hashomer, Israel

4: Laboratoire de Psychologie Medicale, Universitè Libre de Bruxelles and Psy Pluriel, Centre Européen de Psychologie Medicale, Brussels

5: Imperial College School of Medicine, London, UK

6: Laboratory of Biology of Neurodegenerative Disorders, Neuroscience Department, Istituto di Ricerche Farmacologiche Mario Negri IRCCS, Milan, Italy

7: Department of Psychiatry, Athens University Medical School, Athens, Greece

8: University Clinic for Psychiatry, Psychotherapy and Psychosomatic, Martin-Luther-University Halle-Wittenberg, Germany

9: Universite´ Libre de Bruxelles

10: Department of Psychiatry, Dalhousie University, Halifax, Nova Scotia, Canada

11. Department of Biomedical and NeuroMotor Sciences, University of Bologna, Italy

Table of contents

Supplementary Methods………………………………………………………….. p. 2
Supplementary Table 1…………………………………………………………… p. 6
Supplementary Table 2…………………………………………………………… p. 7
Supplementary Table 3…………………………………………………………… p. 10
Supplementary Table 4…………………………………………………………… p. 10
Supplementary Table 5…………………………………………………………… p. 10
Supplementary Table 6…………………………………………………………… p. 11
Supplementary Table 7…………………………………………………………… p. 13
Supplementary Table 8…………………………………………………………… p. 15
Supplementary Table 9…………………………………………………………… p. 17
Supplementary Table 10………………………………………………………….. p. 20
Supplementary Table 11………………………………………………………….. p. 22
Supplementary Table 12………………………………………………………….. p. 22
Supplementary Figure 1………………………………………………………….. p. 24
Supplementary Figure 2………………………………………………………….. p. 24
Supplementary Figure 3………………………………………………………….. p. 25
Supplementary Figure 4………………………………………………………….. p. 25
Supplementary Figure 5………………………………………………………….. p. 26
Supplementary Figure 6………………………………………………………….. p. 27
Supplementary Figure 7………………………………………………………….. p. 30
References………………………………………………………………………… p. 30

Supplementary Methods

DNA extraction in GSRD

Genomic DNA was extracted from whole blood with an automated nucleic acid extractor (Maxwell, Promega, US). Quantity and quality were checked by a nanoscale spectrophotometer and for samples having a 260/280 absorbance ratio below 1.2 the extraction process was repeated.

Whole exome sequencing, genotyping and quality control in GSRD

The initial quality control was performed using the FASTQ pipeline (1). Trimming of the reads with barcode sequences was performed with fastx trimmer from the fastx toolkit (2). To align the trimmed reads to the reference human genome (human assembly GRCh37/hg19) we used TopHat2 (3) with the parameters mate-inner-dist=118 for the paired reads, and mate-std-dev=52.

Variants were called using FreeBayes, a Bayesian genetic variant detector designed to find SNPs (single-nucleotide polymorphisms), indels (insertions and deletions), MNPs (multi-nucleotide polymorphisms), and complex events (composite insertion and substitution events). FreeBayes is haplotype-based, in the sense that it calls variants based on the literal sequences of reads aligned to a particular target, not their precise alignment. This method avoids one of the core problems with alignment-based variant detection that identical sequences may have multiple possible alignments (4). Poorly called variants were pruned out (quality score < 30, leaving in only variants with base call accuracy of 99.9% (5)) and variants with read depth < 10 (6) were excluded. Variants with ≥ 2% missing rate, indels > 5 bp and SNPs within 3 bp from an indel (7), variants without at least two reads balanced to each side of the site (RPR < 1 or RPL < 1) or without reads on both strands (SAF=0 or SAR=0) (8) were also excluded. We excluded subjects having genotyping rate ≤ 95%, gender discrepancies, cryptic relatedness (identity by descent (IBD)>0.1875(9)), abnormal heterozygosity and population outliers (outside five standard deviations from the mean for the first 20 population principal components). After quality control, mean read depth was 79.82 (SD 62.15). Indels were realigned using left-normalization, and multiallelic variants were split into individual VCF lines using BCFtools (https://samtools.github.io/bcftools/bcftools.html).

Population principal components were calculated on a linkage-disequilibrium-pruned set of variants with MAF ≥ 0.02. Indeed it has been demonstrated that the inclusion of low frequency or rare variants does not improve the detection of population stratification (10).

Quality control of genome-wide data was performed in line with the criteria used for the exome sequence data and a previous study on this sample (11). Genome-wide data was imputed using the Michigan imputation server and the Haplotype Reference Consortium (HRC, version r1.1 2016) as reference panel (12). Only common variants (MAF > 0.02) were extracted from imputed data and pruned for poor imputation quality (R^2<0.30) (13).

The total number of rare and common variants shared between the exome sequence data and genome-wide data was 161,130 and 129,610, respectively; 120,632 (74.9%) of the rare ones were imputed (in the array data) and 40,497 (25.1%) of them were sequenced/genotyped; 114,198 (88%) of the common variants were imputed (in the array data) and 15,412 were sequenced/genotyped. The shared variants were used to exclude samples with poor concordance as explained in the main manuscript (paragraph 3. Results).

Variant annotation and exome risk score in GSRD

We classified variants into a high functional group and intermediate functional group according to functional consequence scores from the Sequence Ontology (SO) project (14). The first group

included variants with a functional consequence score ≥ 0.90 (frameshift, stop gained, splice region, splice acceptor, splice donor, coding sequence, start lost, incomplete terminal codon, stop lost) and the second group included variants with a functional consequence score ≥ 0.70 (protein altering, missense, initiator codon, inframe deletion, inframe insertion).

Exome risk scores were calculated using different weighting methods for comparison purpose (Eigen, CADD and SO functional scores) as described in paragraph 2.4.2. of the main manuscript. Eigen and CADD score were estimated using predictive modelling based on a wide range of variant annotations, describing variant risk of damaging effects based on conservation, impact on protein function, structure, gene transcription, chromatin structure and others. Eigen scores differently from CADD scores were calculated using unsupervised machine learning, meaning that variants were not labelled as benign or damaging in the training set, but this distinction was figured out by the model based on a number of variant annotations (15). When using Eigen and CADD score, we extracted the raw scores and re-scaled them between 1-25 (to not have negative values). The used weights based on SO functional scores are reported in Box 1. When we used SO functional scores, gene scores were calculated using GeneVarAssoc and getVarScores software (16). For scores based on common variants, we created clumps around variants prioritized according to their functional scores (the variant with the highest functional score was kept), using a R^2 threshold of 0.5 and a window size of 30 Kbp. This approach was used instead of the classic clumping based on association p values because the scores we calculated were substantially based on variant functional consequences and not on the difference in frequency between cases and controls.

Box 1

- anyVariant 20
- maybeRegulatoryOrIntron 60
- alsoUTR 65
- coding 70
- nonsynonymous 90
- LOF 95
- polyphenProbablyOrPossiblyDamaging 95
- SIFTDeleterious 100

In terms of included gene sets, we downloaded Gene Ontology (GO), Reactome, Biocarta, KEGG and other canonical gene sets (pathway interaction database and signal transduction pathways) from the Molecular Signatures Database (MSigDB version 6.2) (17). In addition, we included gene sets from SynaptomeDB (18), gene sets previously associated with major depressive disorder (MDD) (19), schizophrenia (20) (21) or bipolar disorder (22). A total of 7266 gene sets and 18908 genes were considered. In order to avoid bigger genes from driving most of the effect of a gene set, we added a further weight ($\omega_{size,m}$) that reduced the score of a gene as a function of its number of variants ($\omega_{size,m}$ = $1+(s_m - \min\{s_i\})/(\max\{s_i\} - \min\{s_i\})$, where s_m: number of variants in a gene, min{si}: number of variants in the smallest gene, max{si}: number of variants in the biggest gene) (23).

Replication samples: STAR*D and GENDEP

The Sequenced Treatment Alternatives to Relieve Depression (STAR*D) study was a NIMH-funded study aimed to determine the effectiveness of different treatments for patients with MDD who have not responded to the first antidepressant treatment. Non-psychotic MDD (DSM-IV criteria) patients with age between 18 and 75 years were enrolled from primary care or psychiatric outpatient clinics. Severity of depression was assessed using the 16-item Quick Inventory of Depressive Symptomatology-Clinician Rated (QIDS-C16) at baseline, weeks 2, 4, 6, 9, and 12, while HRSD–17 was administered at each level entry and exit. All patients received citalopram in level 1. Participants without sufficient symptomatic benefit were eligible for randomization to level 2 treatments, which entail four switch options (sertraline, bupropion, venlafaxine, cognitive therapy) and three citalopram augment options (bupropion, buspirone, cognitive therapy). 1953 patients were included in the genetic study. Detailed description of the study design and population are reported elsewhere (24).

The Genome-Based Therapeutic Drugs for Depression (GENDEP) project was a 12-week partially randomized open-label pharmacogenetic study with two active treatment arms. 867 patients with unipolar depression (ICD-10 or DSM-IV criteria) aged 19–72 years were recruited at nine European centres. Eligible participants were allocated to flexible-dosage treatment with either escitalopram (10–30 mg daily, 499 subjects) or nortriptyline (50–150 mg daily, 368 subjects). Severity of depression was assessed weekly by the Montgomery-Asberg Depression Rating Scale (MADRS), Hamilton Rating Scale for Depression (HRSD–17) and Beck Depression Inventory (BDI). Detailed information about the GENDEP study has been previously reported (25).

In STAR*D longitudinal data referred to level 1 and level 2 were used to create the phenotypes. Response was defined as a QIDS-C16 < 13 [equivalent to MADRS of 22] and score decrease of at least 50% compared to baseline at level 1 exit, while TRD was defined as lack of response at level 2 exit. In GENDEP prospective data collected during the 12-week trial were combined with the retrospective information on previous antidepressant treatments of the current episode to determine the phenotypes, as described in a previous study (26).

Genome-wide data in STAR*D were obtained using Affymetrix Human Mapping 500K Array Set in 969 subjects and Affymetrix Genome-Wide Human SNP Array 5.0 (Affymetrix, South San Francisco, California) in the remaining 979 samples, while in GENDEP Illumina Human610-quad bead chip (Illumina, Inc., San Diego) was used (25) (27). Further genotyping in both samples was performed by the Illumina Infinium Exome-24 v1.0 BeadChip that includes ~ 250K variants. Pre-imputation quality control was performed according to the following criteria: 1) variants with missing rate ≥ 5%; 2) monomorphic variants; 3) subjects with genotyping rate < 97%; 4) subjects with gender discrepancies; 5) subjects with abnormal heterozygosity; 6) related subjects (identity by descent (IBD) >0.1875 (9)); 7) population outliers according to Eigensoft analysis of linkage-disequilibrium-

pruned genetic data (28); 8) GWAS-exome discordant and 9) non-white subjects in STAR*D. The number of included subjects after quality control and their clinical-demographic features are reported in Supplementary Table 12.

Gene- and pathway-based scores were calculated in STAR*D and GENDEP following the same approach used in GSRD. Rare and common variants were distinguished based on the MAF threshold $1/\sqrt{(2n)}$ where n was the sample size (29). Scores were adjusted for the same covariates used in the GSRD sample (population principal components and centre of recruitment). In GSRD and replication samples the scores were standardized to allow comparability.

Clinical risk scores were calculated in the replication samples using the same approach applied in GSRD, using the effect sizes obtained in GSRD training sample. In GENDEP there were no subjects with chronic depression according to the DSM-IV definition (duration of the episode of at least two years), thus we used 1 year as threshold to define chronic depression since there is some evidence that outcome is poorer after 1 year (30). The maximum number of depressive episodes in GENDEP was 3, thus we used 2 (3rd quantile) as threshold instead of 4 (which corresponded to the 3rd quantile in GSRD sample). In STAR*D the MADRS scale was not available, thus we used QIDS-C16 item 11 (view of myself) and item 12 (suicidality) to calculate the pessimism score and items 10 (concentration), 13 (general interest), 14 (energy level) and 15 (feeling slow down) to calculate the interest activity score.

Supplementary Table 1: clinical-demographic characteristics of patients included in the training sample (n=847) and testing sample (n=362). Baseline MADRS refers to the beginning of the current depressive episode. BMI=body max index. MADRS=Montgomery and Åsberg Depression Rating Scale. TRD=treatment-resistant depression. Mean ± standard deviation is reported for continuous variables and distribution for dichotomous ones. T tests, chi2 or Fisher's exact tests were used to calculate p values.

Variable	Training sample (n=847)	Testing sample (n=362)	p value
Age	51.44±13.94	51.87±14.16	0.63
Gender (F/M)	566/281	235/127	0.56
Education	Primary school n=59<br>Secondary school n=309<br>High school n=219<br>University n=253	Primary school n=29<br>Secondary school n=146<br>High school n=81<br>University n=103	0.43
Work status	Full time n=325<br>Part time n=70<br>Retired n=169<br>Student n=24<br>Unemployed n=215<br>Other n=38	Full time n=137<br>Part time n=38<br>Retired n=77<br>Student n=8<br>Unemployed n=86<br>Other n=13	0.73
Age at onset	36.85±14.95	35.94±15.70	0.37
Number of previous depressive episodes	3.61±2.77	3.77±2.92	0.44
Baseline MADRS score	34.56±7.36	33.85±7.69	0.14
Current MADRS score	24.73±11.13	24.78±11.60	0.95

Melancholic MDD (yes/no)	552/289	247/114	0.38
Psychotic features (yes/no)	40/551	16/244	0.85
Chronic depression (yes/no)	150/307	66/134	1
Current suicidal risk (yes/no)	416/430	176/186	0.91
Generalized anxiety disorder (yes/no)	87/759	41/321	0.66
Phenotype of interest	TRD n=353<br>Non-response n=291<br>Response n=203	TRD n=151<br>Non-response n=125<br>Response n=86	0.99
Treatment	Serotonergic n=421<br>Noradrenergic n=271<br>Serotonergic-noradrenergic n=128<br>Other n=27	Serotonergic n=192<br>Noradrenergic n=93<br>Serotonergic-noradrenergic n=59<br>Other n=18	0.10

Supplementary Table 2: A. clinical variables tested for possible association with TRD (TRD vs. response) and non-response (non-response vs. response) in the training sample only for predictor selection. The variables independently associated with the phenotype of interest after Bonferroni correction (alpha=0.0018) are highlighted in red and they were included in the clinical risk score. **B.** Distribution of the clinical-demographic variables across the phenotypic groups in the training sample. **C.** Distribution of the clinical-demographic variables across the phenotypic groups in the testing sample. MADRS=Montgomery and Åsberg Depression Rating Scale. MADRS subscales were defined according to the previous literature and they were tested because of their previously found association with antidepressant response (31).

A. Statistics in the training sample

Variable	TRD vs. response p value	Non-response vs. response p value
Suicidality yes/no	6.32e-10	2.59e-06
Number of previous episodes	1.49e-04	0.0006
Psychotic depression yes/no	0.01	0.02
Melancholia yes/no	0.76	0.95
Chronic depression yes/no	2.29e-14	8.06e-08
MADRS baseline total	2.23e-04	0.92
MADRS baseline mood	2.5e-06	0.45
MADRS baseline mood-anxiety	6.58e-05	0.41
MADRS baseline anxiety	0.65	0.53
MADRS baseline cognition	7.58e-06	0.55
MADRS baseline pessimism	5.50e-04	0.55
MADRS baseline interest-activity	3.95e-05	0.67
MADRS baseline sleep	4.76e-03	0.48
MADRS baseline appetite	0.07	0.008
MADRS baseline vegetative	0.77	0.15
Age	0.03	0.16
Gender	1	0.58
BMI	0.01	0.26
Marital status	0.73	0.64
Work status	0.01	0.05
Age at onset	0.39	0.03

Duration of current episode	0.003	0.05
Generalized anxiety disorder yes/no	0.39	0.17
Panic disorder yes/no	0.056	0.58
Obsessive-compulsive disorder yes/no	0.67	0.35
Post-traumatic stress disorder yes/no	1	0.84
Thyroid disorder yes/no	0.61	0.23
Other medical illness yes/no	0.18	0.12

B. Description of clinical-demographic variables in the training sample

Variable	TRD (n=353)	Non-response (n=291)	Response (n=203)
Suicidality yes/no	195(55.4%)/157	156(53.6%)/135	65(32%)/138
Number of previous episodes	3.91±2.91	3.80±2.81	2.83±2.27
Psychotic depression yes/no	20(8.8%)/208	17(8.2%)/190	3(1.9%)/153
Melancholia yes/no	233(66.4%)/118	189(65.4%)/100	130(64.7%)/71
Chronic depression yes/no	91(25.8%)/262	49(16.8%)/242	10(4.9%)/193
MADRS baseline total	33.88±6.49	33.65±8.00	33.58±7.54
MADRS baseline mood	12.90±2.03	12.07±2.69	11.88±2.71
MADRS baseline mood-anxiety	16.48±2.65	15.67±3.41	15.41±3.24
MADRS baseline anxiety	3.58±1.11	3.60±1.17	3.53±1.23
MADRS baseline cognition	17.43±3.76	16.02±4.71	15.77±4.45
MADRS baseline pessimism	5.80±2.36	5.19±2.53	5.04±2.55
MADRS baseline interest-activity	11.63±2.16	10.84±2.86	10.73±2.75
MADRS baseline sleep	3.82±1.34	3.57±1.40	3.48±1.47
MADRS baseline appetite	2.23±1.73	2.08±1.67	2.51±1.83
MADRS baseline vegetative	6.05±2.39	5.65±2.44	5.98±2.64
Age	52.43±13.82	51.48±12.79	49.67±15.53
Gender (F/M)	233(66%)/120	199(68.4%)/92	134(66%)/69
BMI	26.01±5.10	25.47±5.09	24.97±4.55
Marital status	Married/living with partner n=176<br>Separated/divorced n=67<br>Single n=88<br>Widowed n=22	Married/living with partner n=137<br>Separated/divorced n=77<br>Single n=64<br>Widowed n=13	Married/living with partner n=99<br>Separated/divorced n=44<br>Single n=51<br>Widowed n=9
Work status	Full time n=133<br>Part time n=21<br>Retired n=86<br>Student n=9<br>Unemployed n=92<br>Other n=12	Full time n=115<br>Part time n=21<br>Retired n=49<br>Student n=6<br>Unemployed n=77<br>Other n=23	Full time n=77<br>Part time n=28<br>Retired n=34<br>Student n=9<br>Unemployed n=46<br>Other n=9
Age at onset	38.55±15.08	34.30±14.28	37.44±15.23
Duration of current episode (days)	249.17±193.47	225.98±202.26	155.82±185.84
Generalized anxiety disorder yes/no	37(10.5%)/315	34(11.7%)/257	16(7.9%)/187
Panic disorder yes/no	25(7.1%)/327	30(10.3%)/261	25(12.3%)/178
Obsessive-compulsive disorder yes/no	3(0.09%)/347	8(2.8%)/280	3(1.5%)/198

Post-traumatic stress disorder yes/no	5(1.4%)/347	5(1.7%)/286	3(1.5%)/200
Thyroid disorder yes/no	51(14.5%)/301	38(13.1%)/253	34(17%)/166
Other medical illness yes/no	152(43.1%)/201	128(44%)/163	75(36.9%)/128

C. Description of clinical-demographic variables in the testing sample

Variable	TRD (n=151)	Non-response (n=125)	Response (n=86)
Suicidality yes/no	88(58.3%)/63	57(45.6%)/68	31(36%)/55
Number of previous episodes	4.01±3.24	3.59±2.33	3.58±3.06
Psychotic depression yes/no	11(7.3%)/96	3(2.4%)/80	2(2.3%)/68
Melancholia yes/no	102(68%)/48	87(69.6%)/38	58(67.4%)/28
Chronic depression yes/no	47(31.1%)/104	17(13.6%)/108	2(2.3%)/84
MADRS baseline total	35.98±7.23	32.66±7.86	31.84±7.40
MADRS baseline mood	12.97±2.36	11.82±2.43	11.48±2.77
MADRS baseline mood-anxiety	16.56±2.83	15.37±3.09	14.82±3.54
MADRS baseline anxiety	3.59±1.02	3.55±1.22	3.35±1.43
MADRS baseline cognition	17.15±4.13	15.23±4.79	14.92±4.67
MADRS baseline pessimism	5.80±2.24	4.68±2.65	4.67±2.51
MADRS baseline interest-activity	11.35±2.61	10.55±2.96	10.25±2.87
MADRS baseline sleep	3.66±1.44	3.65±1.20	3.37±1.66
MADRS baseline appetite	2.71±1.65	2.15±1.60	2.01±1.75
MADRS baseline vegetative	6.37±2.32	5.81±2.29	5.38±2.78
Age	51.89±14.00	50.14±12.99	54.35±15.79
Gender (F/M)	92(60.9%)/59	90(72.0%)/35	235(64.9%)/127
BMI	25.91±5.36	24.62±4.43	25.72±5.24
Marital status	Married/living with partner n=79<br>Separated/divorced n=26<br>Single n=35<br>Widowed n=11	Married/living with partner n=71<br>Separated/divorced n=17<br>Single n=32<br>Widowed n=5	Married/living with partner n=49<br>Separated/divorced n=13<br>Single n=20<br>Widowed n=4
Work status	Full time n=63<br>Part time n=14<br>Retired n=33<br>Student n=2<br>Unemployed n=34<br>Other n=5	Full time n=49<br>Part time n=15<br>Retired n=19<br>Student n=1<br>Unemployed n=35<br>Other n=4	Full time n=25<br>Part time n=9<br>Retired n=25<br>Student n=5<br>Unemployed n=17<br>Other n=5
Age at onset	37.43±14.97	32.14±14.93	38.86±17.05
Duration of current episode (days)	240.04±188.97	183.21±162.00	165.35±183.69
Generalized anxiety disorder yes/no	20(13.2%)/131	17(13.6%)/108	4(4.7%)/82
Panic disorder yes/no	9(6%)/142	14(11.2%)/111	7(8.1%)/79
Obsessive-compulsive disorder yes/no	2(1.3%)/148	0/121	1(1.2%)/85
Post-traumatic stress disorder yes/no	2(1.3%)/149	1(0.8%)/124	1(1.2%)/85
Thyroid disorder yes/no	23(15.2%)/128	19(15.2%)/106	15(18%)/69
Other medical illness yes/no	60(39.7%)/91	53(42.4%)/72	44(51.2%)/42

Supplementary Table 3: number of variants available after quality control in the GSRD sample. SNP=single nucleotide polymorphism; insdel=insertion/deletions; MNPs=multi-nucleotide polymorphisms. SO=sequence ontology project. The number of variants with SO functional score ≥0.90 was lower than the number of deleterious and damaging variants according to SIFT and Polyphen scores, but only the 73% of variants in the first group had a MAF<0.02 while 85% in both the other two groups.

**Variant type**	**All**	**MAF<0.02**	**SO functional score >=0.90**	**SIFT deleterious**	**Polyphen damaging**
SNP	1,134,094	996,390	35,150	89,810	106,962
Insdel	85,944	75,483	7,263	-	-
MNPs	9,097	7,874	256	437	471
Other	1,615	1,462	167	-	-
Total	1,230,750	1,081,209	42,836	90,247	107,433

Supplementary Table 4: exome-wide distribution of variants with SO functional score ≥ 0.90 or ≥ 0.70, SIFT deleterious and PolyPhen damaging/probably damaging variants among phenotypic groups. The mean number of alternative alleles and SD are reported in each phenotypic group.

Functional group	**TRD**	**Non-response**	**Response**	**TRD vs. resp.**	**TRD vs. non-resp. vs. response**
SO functional score ≥ 0.90	2350.41±42.55	2352.5±52.24	2347.98±39.61	E= -0.002, SE=0.002, z= -0.81, p=0.42	E= 8.20e-05, SE=0.0006, t=0.13, p=0.89
SO functional score ≥ 0.70	9854.19±134.44	9867.95±170.07	9854.35±129.58	E=0.001, SE=0.0008, z=1.45, p=0.15	E= -0.0003, SE=0.0002, t=-1.39, p=0.16
SIFT deleterious	1481.04±35.92	1484.33±39.27	1483.87±36.60	E=0.004, SE=0.002, z=1.82, p=0.07	E= -0.001, SE=0.0007, t=-1.44, p=0.15
PolyPhen damaging	1730.02±40.39	1730.82±46.68	1727.78±38.98	E=0.0002, SE=0.002, z=0.10, p=0.92	E=0.0002, SE=0.0006, t=0.29, p=0.77

Supplementary Table 5: distribution of variants with SO functional score ≥ 0.90 per gene among phenotypic groups. The number of subjects carrying at least one alternative allele in a certain gene is also reported. For 14353 genes we observed at least one variant with SO functional score ≥ 0.90. Only genes with p<1e-03 are reported.

**TRD vs. response**				
**Gene**	**TRD** n=504	**Non-response** n=416	**Response** n=289	**Statistics**
ADGB	0.62±0.65<br>>=1 var. 266 (52.8%)	0.54±0.65<br>>=1 var. 189 (45.4%)	0.45±0.54<br>>=1 var. 123 (42.6%)	E= -0.52, SE=0.13, z=-3.85, p=1.1e-04
KIF18B	0.06±0.25<br>>=1 var. 30 (6%)	0.10±0.30<br>>=1 var. 41 (9.9%)	0.14±0.36<br>>=1 var. 39 (13.5%)	E=0.89, SE=0.26, z=3.38, p=7.15e-04
SMC1B	0.90±0.74<br>>=1 var. 339 (67%)	0.96±0.72<br>>=1 var. 299 (71.9%)	1.09±0.71<br>>=1 var. 228 (78.9%)	E=0.37, SE=0.11, z=3.381, p=7.2e-04
**TRD - non-resp - response**				

Gene	TRD	Non-response	Response	Statistics
WDR90	0.042±0.20<br>>=1 var 21 (4.1%)	0.0096±0.10<br>>=1 var 4 (0.96%)	0.0069±0.08<br>>=1 var 2 (0.69%)	E= 0.62, SE=0.15, t=4.16, p=3.44e-05
KIF18B	0.06±0.25<br>>=1 var. 30 (6%)	0.10±0.30<br>>=1 var. 41 (9.9%)	0.14±0.36<br>>=1 var. 39 (13.5%)	E= -0.29, SE=0.07, t=-3.90, p=1.006e-04
FAM169B	2.00±0.04<br>>2 var 1 (0.2%)	2.00±0.12<br>>2 var 3 (0.7%)	1.97±0.17<br>>2 var 0	E= 0.71, SE=0.19, t=3.69, p=2.35e-04
SMC1B	0.90±0.74<br>>=1 var. 339 (67%)	0.96±0.72<br>>=1 var. 299 (71.9%)	1.09±0.71<br>>=1 var. 228 (78.9%)	E= -0.11, SE=0.03, t=-3.62, p=3.04e-04
ENPP2	0	0.007±0.08<br>>=1 var 3 (0.7%)	0.28±0.18<br>>=1 var 7 (2.4%)	E= -0.77, SE=0.21, t= -3.61, p=3.22e-04
ALDH3A1	0.008±0.09<br>>=1 var 4 (0.8%)	0.19±0.14<br>>=1 var 8 (1.9%)	0.04±0.21<br>>=1 var 13 (4.5%)	E= -0.55, SE=0.15, t= -3.56, p=3.90e-04
ADGB	0.62±0.65<br>>=1 var. 266 (52.8%)	0.54±0.65<br>>=1 var. 189(45.4%)	0.45±0.54<br>>=1 var. 123 (42.6%)	E= 0.12, SE=0.03, t=3.49, p=4.95e-04
LOC101929680*	0.41±0.63<br>>=1 var 169 (33.5%)	0.21±0.54<br>>=1 var 108 (26%)	0.26±0.51<br>>=1 var 70 (24.3%)	E= 0.12, SE=0.04, t=3.40, p=7.00e-04
SCN9A*	0.41±0.63<br>>=1 var 169 (33.5%)	0.21±0.54<br>>=1 var 108 (26%)	0.26±0.51<br>>=1 var 70 (24.3%)	E= 0.13, SE=0.04, t=3.40, p=7.00e-04
KCNIP4	1.60±0.99<br>>=1 var 435 (86.3%)	1.44±0.99<br>>=1 var 399 (96%)	1.40±0.90<br>>=1 var 243 (84%)	E=0.08, SE=0.02, t=3.34, 8.74e-04

* SCN1A and SCN9A antisense RNA 1, its position mostly overlaps with SCN9A

Supplementary Table 6: distribution per gene of variants predicted to be deleterious according to SIFT score in **A** (16483 genes had at least one of these variants) and damaging according to PolyPhen score in **B** (16947 genes had at least one of these variants) among phenotypic groups. The number of subjects carrying at least one alternative allele in a certain gene is also reported. Only genes with p<1e-03 are reported.

A

**TRD vs. response**				
**Gene**	**TRD** n=504	**Non-response** n=416	**Response** n=289	**Statistics**
FLG	0.66±1.08<br>>=1 var 156 (31%)	0.82±1.14<br>>=1 var 166 (40%)	0.96±1.29<br>>=1 var 121 (42%)	E=0.24, SE=0.07, z=3.51, p=4.46e-04
LOC101927267*	0.04±0.19<br>>=1 var 19 (3.8%)	0.06±0.25<br>>=1 var 25 (6%)	0.09±0.30<br>>=1 var 25 (8.7%)	E=1.14, SE=0.33, z=3.46, p=5.42e-04
CR2	0.67±0.71<br>>=1 var 273 (54%)	0.61±0.68<br>>=1 var 210 (50%)	0.51±0.66<br>>=1 var 121 (42%)	E=-0.41, SE=0.12, z=-3.40, p=6.70e-04
PRPH*	0.04±0.19<br>>=1 var 18 (3.6%)	0.06±0.25<br>>=1 var 24 (5.8%)	0.09±0.29<br>>=1 var 24 (8.3%)	E=1.15, SE=0.34, z=3.38, p=7.14e-04
CHKB-CPT1B	0.01±0.11<br>>=1 var 6 (1.2%)	0.02±0.13<br>>=1 var 7 (1.7%)	0.04±0.20<br>>=1 var 12 (4.2%)	E=1.71, SE=0.51, z=3.32, p=8.97e-04
**TRD - non-resp - response**				
**Gene**	**TRD**	**Non-response**	**Response**	**Statistics**
EPX	0.68±0.68<br>>=1 var 282 (56%)	0.57±0.63<br>>=1 var 205 (49.3%)	0.53±0.65<br>>=1 var 129 (44.6%)	E=0.13, SE=0.03, t=3.88, p=1.11 e-04
FLG	0.66±1.08<br>>=1 var 156 (31%)	0.82±1.14<br>>=1 var 166 (40%)	0.96±1.29<br>>=1 var 121 (42%)	E=-0.07, SE=0.02, t=-3.76, p=1.78e-04
SLC35E4	0	0.01±0.011<br>>=1 var 5 (1.2%)	0.03±0.16<br>>=1 var 8 (2.8%)	E=-0.77, SE= 0.21, t=-3.63, p=2.92e-04
ABCA2	0.002±0.04<br>>=1 var 1 (0.2%)	0.02±0.14<br>>=1 var 8 (1.9%)	0.03±0.16<br>>=1 var 8 (2.8%)	E=-0.67, SE=0.19, t=-3.60, p=3.31e-04
MIR3654	0.02±0.15<br>>=1 var 12 (2.4%)	0.04±0.19<br>>=1 var 16 (3.8%)	0.07±0.27<br>>=1 var 20 (6.9%)	E=-0.38, SE=0.11, t=-3.51, p=4.61e-04
TUT1	0.02±0.15<br>>=1 var 12 (2.4%)	0.03±0.18<br>>=1 var 14 (3.4%)	0.07±0.27<br>>=1 var 20 (6.9%)	E=-0.39, SE=0.11, t=-3.51, p=4.69e-04

SRR	0.10±0.31<br>>=1 var 48 (9.5%)	0.08±0.28<br>>=1 var 35 (8.4%)	0.04±0.19<br>>=1 var 11 (3.8%)	E=0.28, SE=0.08, t=3.47, p=5.47e-04
ACVRL1	0	0	0.02±0.13<br>>=1 var 5 (1.7%)	E=-1.18, SE=0.34, t=-3.44, p=5.97e-04
PRPH*	0.04±0.19<br>>=1 var 18 (3.6%)	0.06±0.25<br>>=1 var 24 (5.8%)	0.09±0.29<br>>=1 var 24 (8.3%)	E=-0.32, SE=0.09, t=-3.39, p=7.28e-04
LOC101927267*	0.04±0.19<br>>=1 var 19 (3.8%)	0.06±0.25<br>>=1 var 25 (6%)	0.09±0.30<br>>=1 var 25 (8.7%)	E=-0.31, SE=0.09, t=-3.36, p=7.94e-04

* LOC101927267 and PRPH show substantial position overlap

B

**TRD vs. response**				
**Gene**	**TRD** n=504	**Non-response** n=416	**Response** n=289	**Statistics**
LOC101927267	0.04±0.19<br>>=1 var 19 (3.8%)	0.06±0.25<br>>=1 var 24 (5.8%)	0.09±0.30<br>>=1 var 25 (8.7%)	E=1.14, SE=0.33, z=3.46, p=5.42e-04
PRPH	0.04±0.19<br>>=1 var 18 (3.6%)	0.06±0.25<br>>=1 var 24 (5.8%)	0.09±0.29<br>>=1 var 24 (8.3%)	E=1.15, SE=0.34, z=3.38, p=7.14e-04
EPX	0.69±0.69<br>>=1 var 284 (56%)	0.58±0.63<br>>=1 var 209 (50%)	0.54±0.65<br>>=1 var 130 (45%)	E=-0.40, SE=0.12, z=-3.31, p=9.46e-04
MUC5B	0.79±1.15<br>>=1 var 221 (44%)	0.87±1.21<br>>=1 var 190 (45.7%)	1.10±1.34<br>>=1 var 153 (53%)	E=0.21, SE=0.06, z=3.30, p=9.82e-04
**TRD vs. non-resp vs. response**				
**Gene**	**TRD**	**Non-response**	**Response**	**Statistics**
EPX	0.69±0.69<br>>=1 var 284 (56.3%)	0.58±0.63<br>>=1 var 209 (50.2%)	0.54±0.65<br>>=1 var 130 (45%)	E=0.13, SE=0.03, t=3.92, p=9.39e-05
PAGE1	0.008±0.09<br>>=1 var 4 (0.8%)	0.03±0.17<br>>=1 var 13 (3.1%)	0.06±0.29<br>>=1 var 13 (4.5%)	E=-0.43, SE=0.12, t=-3.58, p=3.62e-04
COL5A3	0.006±0.08<br>>=1 var 3 (0.6%)	0.02±0.15<br>>=1 var 9 (2.2%)	0.05±0.23<br>>=1 var 13 (4.5%)	E=-0.51, SE=0.15, t=-3.51, p=4.61e-04
SRR	0.10±0.31<br>>=1 var 48 (9.5%)	0.08±0.28<br>>=1 var 35 (8.4%)	0.04±0.19<br>>=1 var 11 (3.8%)	E=0.28, SE=0.08, t= 3.47, p=5.47e-04
LOC101927267	0.04±0.19<br>>=1 var 19 (3.8%)	0.06±0.25<br>>=1 var 24 (5.8%)	0.09±0.30<br>>=1 var 25 (8.7%)	E=-0.32, SE=0.09, t=-3.44, p=5.91e-04
OR4K5	0.02±0.14<br>>=1 var 10 (2%)	0.05±0.22<br>>=1 var 18 (4.3%)	0.06±0.24<br>>=1 var 17 (5.9%)	E=-0.39, SE=0.11, t=-3.44, p=5.92e-04
ENTPD8	0.02±0.15<br>>=1 var 11 (2.2%)	0.007±0.08<br>>=1 var 3 (9.7%)	0	E=0.70, SE=0.21, t=3.43, p=6.19e-04
MUC5B	0.79±1.15<br>>=1 var 221 (43.8%)	0.87±1.21<br>>=1 var 190 (45.7%)	1.10±1.34<br>>=1 var 153 (53%)	E=-0.06, SE=0.02, t=-3.42, p=6.57e-04
OGDH	0.04±0.20<br>>=1 var 20 (4%)	0.01±0.12<br>>=1 var 6 (1.4%)	0.003±0.06<br>>=1 var 1 (0.3%)	E=0.51, SE=0.15, t=3.40, p=6.85e-04
PRPH	0.04±0.19<br>>=1 var 18 (3.6%)	0.06±0.25<br>>=1 var 24 (5.8%))	0.09±0.29<br>>=1 var 24 (8.3%)	E=-0.32, SE=0.09, t=-3.39, p=7.28e-04
PLVAP	0.002±0.04<br>>=1 var 1 (0.2%)	0.005±0.07<br>>=1 var 2 (0.5%)	0.02±0.15<br>>=1 var 7 (2.4%)	E=-0.81, SE=0.24, t=-3.33, p=8.88e-04
PTPRA	0.05±0.23<br>>=1 var 27 (5.4%)	0.04±0.19<br>>=1 var 15 (3.6%)	0.01±0.10<br>>=1 var 3 (1%)	E=0.38, SE=0.12, t=3.30, p=9.96e-04

Supplementary Table 7: association analyses of Eigen-weighted gene scores with the phenotypic groups. No association survived after Bonferroni correction (n of genes analysed: 21136). Only results with p<1e-03 are shown.

**Whole sample, only rare variants**				
**TRD vs. response**				
**Gene**	**E**	**SE**	**z**	**p**
NBN	0.005	0.001	4.0	6.40e-05
IDH1	0.007	0.002	3.80	1.46e-04
ZNF418	0.009	0.002	3.67	2.43e-04
KRT19P2	0.009	0.002	3.64	2.78e-04
UBXN11	0.005	0.001	3.62	2.94e-04
PHF20L1	0.004	0.001	3.60	3.24e-04
OR4K5	0.009	0.003	3.421	6.25e-04
RORC	0.005	0.001	3.42	6.31e-04
LRP1	-0.003	0.0008	-3.37	7.39e-04
NODAL	0.009	0.003	3.37	7.49e-04
ELF3	0.005	0.002	3.35	8.16e-04
TOMM34	-0.007	0.002	-3.32	8.94e-04
FAM110A	-0.009	0.003	-3.30	9.51e-04
**TRD vs. non-resp vs. response**				
**Gene**	**E**	**SE**	**t**	**p**
NBN	-0.0015	0.0004	-4.10	4.34e-05
ZNF418	-0.0024	0.0006	-4.06	5.18e-05
KRT19P2	-0.0025	0.0006	-3.93	9.04e-05
SCML4	-0.0013	0.0004	-3.75	1.84e-04
IDH1	-0.0019	0.0005	-3.74	1.93e-04
OR4K5	-0.0024	0.0007	-3.64	2.86e-04
UBXN11	-0.0014	0.0004	-3.57	3.65e-04
KATNA1	-0.0019	0.0006	-3.45	5.89e-04
WFDC10B	-0.0042	0.0012	-3.44	5.93e-04
NODAL	-0.0028	0.0008	-3.40	7.01e-04
ELF3	-0.0014	0.0004	-3.38	7.46e-04
VAV2	-0.0007	0.0002	-3.37	7.74e-04
GLUD1	0.0030	0.0009	3.37	7.75e-04
RORC	-0.0013	0.0004	-3.35	8.41e-04
SLPI	0.0019	0.0006	3.34	8.61e-04
**Subsample treated with serotonergic antidepressants, only rare variants**				
**TRD vs. response**				
**Gene**	**E**	**SE**	**t**	**p**
SUMF1	0.009	0.002	3.54	4.00e-04
LYPD6B	0.007	0.002	3.41	6.56e-04
PTPRU	0.007	0.002	3.40	6.83e-04
TEX36-AS1	0.015	0.005	3.38	7.15e-04
ZNF418	0.012	0.004	3.32	8.94e-04
GZMM	-0.014	0.004	-3.32	8.98e-04
TRHR	0.001	0.0029	3.31	9.40e-04
**TRD vs. non-resp vs. response**				
**Gene**	**E**	**SE**	**t**	**p**
ZBTB32	-0.0044	0.0011	-3.94	9.15e-05
UBXN11	-0.0018	0.0005	-3.61	3.27e-04
TAF1A	0.0034	0.0010	3.55	4.11e-04
CHRDL2	-0.0012	0.0004	-3.55	4.11e-04
ZNF418	-0.0030	0.0009	-3.55	4.13e-04
SMYD1	0.0015	0.0004	3.54	4.26e-04
GATS	-0.0030	0.0008	-3.54	4.29e-04
LYPD6B	-0.0020	0.0006	-3.52	4.58e-04
TTC23L	0.0018	0.0005	3.49	5.26e-04
IFNA4	-0.0053	0.0015	-3.46	5.79e-04

KRT19P2	-0.0031	0.0009	-3.45	5.91e-04
HERC6	0.0021	0.0006	3.44	6.18e-04
EFHC1	-0.0023	0.0007	-3.43	6.49e-04
PTPRU	-0.0018	0.0005	-3.42	6.68e-04
TMEM67	-0.0017	0.0005	-3.42	6.71e-04
SLPI	0.0026	0.0008	3.39	7.33e-04
SCML4	-0.0019	0.0006	-3.39	7.45e-04
CCDC8	0.0044	0.0013	3.36	8.28e-04
TEX36-AS1	-0.0042	0.0013	-3.34	8.76e-04
PTPRT	0.0009	0.0003	3.32	9.52e-04
**Subsample treated with noradrenergic antidepressants, only rare variants**				
**TRD vs. response**				
**Gene**	**E**	**SE**	**t**	**p**
IFT80	0.01	0.003	3.34	8.24e-04
RPGRIP1L	0.01	0.003	3.33	8.60e-04
**TRD vs. non-resp vs. response**				
**Gene**	**E**	**SE**	**t**	**p**
KCNK15	-0.005	0.001	-3.93	1.04e-04
RNF144A	0.003	0.0008	3.64	3.16e-04
CCNT2-AS1	-0.004	0.001	-3.60	3.64e-04
RPGRIP1L	-0.003	0.0007	-3.56	4.23e-04
ACMSD	-0.004	0.001	-3.47	5.80e-04
CLK1	-0.002	0.0007	-3.40	7.49e-04
PRR23A	-0.005	0.002	-3.40	7.50e-04
COTL1	0.003	0.0009	3.37	8.37e-04
GDPD5	-0.002	0.0006	-3.33	9.76e-04
**Whole sample, rare + common variants**				
**TRD vs. response**				
**Gene**	**E**	**SE**	**t**	**p**
NBN	0.0051	0.0012	4.16	3.14e-05
ZNF418	0.0094	0.0024	3.91	9.34e-05
NODAL	0.0103	0.0028	3.71	2.10e-04
IDH1	0.0064	0.0018	3.65	2.63e-04
KRT19	0.0086	0.0024	3.58	3.48e-04
RORC	0.0052	0.0015	3.53	4.17e-04
UBXN11	0.0050	0.0014	3.51	4.42e-04
SEC22A	0.0088	0.0025	-3.51	4.43e-04
NDC1	0.0047	0.0014	3.42	6.19e-04
ELF3	0.0051	0.0015	3.37	7.60e-04
GALE	0.0070	0.0021	3.36	7.77e-04
LRP1	-0.0028	0.0008	-3.36	7.81e-04
PAQR5	0.0041	0.0012	3.35	7.95e-04
**TRD vs. non-resp vs. response**				
**Gene**	**E**	**SE**	**t**	**p**
ZNF418	-0.003	0.0006	-4.30	1.86e-05
NBN	-0.001	0.001	-4.29	1.91e-05
KRT19	-0.003	0.0006	-3.91	9.93e-05
NODAL	-0.003	0.0008	-3.85	1.27e-04
RORC	-0.001	0.0004	-3.49	4.99e-04
UBXN11	-0.001	0.0004	-3.48	5.24e-04
IDH1	-0.002	0.0005	-3.45	5.83e-04
NDC1	-0.001	0.0004	-3.40	6.88e-04
GLUD1	0.003	0.0007	3.40	7.01e-04
SEC22A	0.002	0.0005	3.39	7.31e-04
SCML4	-0.001	0.0004	-3.34	8.63e-04
ELF3	-0.001	0.0004	-3.33	8.96e-04
LINC01220	-0.005	0.001	-3.30	9.81e-04
PAQR5	-0.001	0.003	-3.30	9.86e-04
**Subsample treated with serotonergic antidepressants, rare + common variants**				

TRD vs. response				
**Gene**	**E**	**SE**	**t**	**p**
ZNF418	0.014	0.004	3.74	1.84e-04
PBX3	-0.007	0.002	-3.67	2.40e-04
PTPRU	0.007	0.002	3.55	3.85e-04
TOM1L1	0.007	0.002	3.45	5.70e-04
HIST1H4G	-0.21	0.06	-3.44	5.75e-04
RORC	0.008	0.002	3.42	6.24e-04
R3HDM4	-0.012	0.004	-3.35	8.05e-04
GZMM	-0.015	0.004	-3.35	8.07e-04
ZBTB32	0.020	0.006	3.35	8.21e-04
APBA2	0.005	0.001	3.33	8.78e-04
PAM	0.004	0.001	3.33	8.78e-04
SAMD9L	0.006	0.002	3.32	9.03e-04
**TRD vs. non-resp vs. response**				
**Gene**	**E**	**SE**	**t**	**p**
ZNF418	-0.003	0.0007	-3.95	8.78e-05
ZBTB32	-0.004	0.001	-3.84	1.35e-04
KRT19	-0.003	0.0009	-3.60	3.49e-04
PTPRU	-0.002	0.0005	-3.59	3.52e-04
TOM1L1	-0.002	0.0005	-3.54	4.25e-04
PAPOLA	0.002	0.0006	3.54	4.25e-04
IFNA4	-0.005	0.001	-3.48	5.42e-04
UBXN11	-0.002	0.0005	-3.48	5.43e-04
GATS	-0.003	0.0008	-3.47	5.67e-04
CHRD	-0.001	0.0003	-3.43	6.39e-04
APBA2	-0.001	0.0003	-3.36	8.17e-04
ZNF680	-0.002	0.0005	-3.31	9.79e-04
**Subsample treated with noradrenergic antidepressants, rare + common variants**				
**TRD vs. response**				
**Gene**	**E**	**SE**	**t**	**p**
DLGAP2-AS1	-0.079	0.021	-3.77	1.64e-04
EXTL1	0.013	0.0036	3.66	2.50e-04
RNF144A	-0.016	0.0049	-3.37	7.52e-04
**TRD vs. non-resp vs. response**				
**Gene**	**E**	**SE**	**t**	**p**
KCNK15	-0.005	0.001	-3.76	2.03e-04
BIN1	-0.003	0.0007	-3.70	2.55e-04
COTL1	0.003	0.0008	3.61	3.46e-04
EXTL1	-0.003	0.0008	-3.57	4.03e-04
RNF144A	0.003	0.0008	3.56	4.29e-04
ARHGEF4	0.002	0.0005	3.51	5.16e-04
PRR23A	-0.005	0.0016	-3.43	6.73e-04
CLK1	-0.002	0.0007	-3.35	8.89e-04

Supplementary Table 8: association analyses of Eigen-weighted gene set scores with the phenotypic groups. No association survived after Bonferroni correction (n of gene sets analysed: 7266). Only results with p<1e-03 are shown.

Whole sample, only rare variants				
**TRD vs. response**				
**Gene set**	**E**	**SE**	**z**	**p**
GO AMINO ACID TRANSMEMBRANE TRANSPORTER ACTIVITY	0.0006	0.0002	3.70	2.17e-04
GO DISULFIDE OXIDOREDUCTASE ACTIVITY	0.0009	0.0002	3.55	3.90e-04
GO BETA AMYLOID BINDING	0.0008	0.0002	3.46	5.44e-04
GO L AMINO ACID TRANSMEMBRANE TRANSPORTER ACTIVITY	0.0006	0.0002	3.41	6.55e-04

REACTOME FGFR2C LIGAND BINDING AND ACTIVATION	0.002	0.0005	3.39	6.92e-04
GO REGULATION OF THYMOCYTE AGGREGATION	0.001	0.0003	3.31	9.23e-04
**TRD vs. non-resp vs. response**				
**Gene set**	**E**	**SE**	**t**	**p**
GO DISULFIDE OXIDOREDUCTASE ACTIVITY	-0.0003	6.81e-05	-3.37	7.67e-04
GO AMINO ACID TRANSMEMBRANE TRANSPORTER ACTIVITY	-0.0001	4.12e-05	-3.36	7.99e-04
GO REGULATION OF THYMOCYTE AGGREGATION	-0.0003	8.19e-05	-3.31	9.55e-04
GO MAMMARY GLAND LOBULE DEVELOPMENT	0.0003	8.02e-05	3.31	9.77e-04
**Subsample treated with serotonergic antidepressants, only rare variants**				
**TRD vs. response**				
**Gene set**	**E**	**SE**	**t**	**p**
GO VASCULAR ENDOTHELIAL GROWTH FACTOR RECEPTOR SIGNALING PATHWAY	0.0008	0.0002	3.45	5.59e-04
**TRD vs. non-resp vs. response**				
**Gene set**	**E**	**SE**	**t**	**p**
PID CD40 PATHWAY	-0.0004	1.04e-04	-3.40	7.23e-04
GO GLYCERALDEHYDE 3 PHOSPHATE METABOLIC PROCESS	0.0005	1.52e-04	3.39	7.49e-04
GO VASCULAR ENDOTHELIAL GROWTH FACTOR RECEPTOR SIGNALING PATHWAY	-0.0002	6.13e-05	-3.31	9.91e-04
**Subsample treated with noradrenergic antidepressants, only rare variants**				
**TRD vs. response**				
**Gene set**	**E**	**SE**	**t**	**p**
GO REGULATION OF INSULIN SECRETION INVOLVED IN CELLULAR RESPONSE TO GLUCOSE STIMULUS	0.0012	0.0004	3.57	3.57e-04
GO SUMO TRANSFERASE ACTIVITY	0.0025	0.0007	3.41	5.68e-04
GO TRNA METABOLIC PROCESS	0.0008	0.0002	3.41	6.45e-04
GO RESPONSE TO COCAINE	-0.0015	0.0004	-3.39	6.92e-04
**TRD vs. non-resp vs. response**				
**Gene set**	**E**	**SE**	**t**	**p**
GO RESPONSE TO COCAINE	0.0003	9.33e-05	3.66	2.95e-04
GO I KAPPAB KINASE NF KAPPAB SIGNALING	0.0003	9.55e-05	3.39	7.83e-04
**Whole sample, rare + common variants**				
**TRD vs. response**				
**Gene set**	**E**	**SE**	**t**	**p**
GO AMINO ACID TRANSMEMBRANE TRANSPORTER ACTIVITY	0.0006	1.52e-04	3.68	2.37e-04
GO SYNAPTIC TRANSMISSION DOPAMINERGIC	-0.0015	4.28e-04	-3.40	6.66e-04
GO REGULATION OF CHROMOSOME ORGANIZATION	0.0003	8.30e-05	3.37	7.63e-04
GO L AMINO ACID TRANSMEMBRANE TRANSPORTER ACTIVITY	0.0006	1.84e-04	3.35	8.02e-04
**TRD vs. non-resp vs. response**				
**Gene set**	**E**	**SE**	**t**	**p**
GO REGULATION OF CHROMOSOME ORGANIZATION	-7.54e-05	0.00002	-3.47	5.30e-04
GO SYNAPTIC TRANSMISSION DOPAMINERGIC	3.75e-04	0.0001	3.35	8.35e-04
**Subsample treated with serotonergic antidepressants, rare + common variants**				
**TRD vs. response**				
**Gene set**	**E**	**SE**	**t**	**p**
PID BCR 5PATHWAY	0.0009	0.0002	3.67	2.43e-04
PID CD40 PATHWAY	0.001	0.0004	3.63	2.84e-04
GO RESPONSE TO NICOTINE	0.001	0.0003	3.38	7.22e-04
PID TRAIL PATHWAY	0.002	0.0005	3.37	7.48e-04
GO AMINO ACID TRANSMEMBRANE TRANSPORTER ACTIVITY	0.0007	0.0002	3.32	8.93e-04
**TRD vs. non-resp vs. response**				
**Gene set**	**E**	**SE**	**t**	**p**
PID CD40 PATHWAY	-0.0004	1.03e-04	-4.07	5.28e-05

PID BCR 5PATHWAY	-0.0002	6.12e-05	-3.67	2.65e-04
PID CERAMIDE PATHWAY	-0.0003	7.76e-05	-3.50	4.92e-04
GO GLYCERALDEHYDE 3 PHOSPHATE METABOLIC PROCESS	0.0005	1.51e-04	3.49	5.20e-04
GO G PROTEIN COUPLED GLUTAMATE RECEPTOR SIGNALING PATHWAY	0.0004	1.13e-04	3.45	6.09e-04
GO AMINO ACID TRANSMEMBRANE TRANSPORTER ACTIVITY	-0.0002	5.44e-05	-3.38	7.77e-04
PID TRAIL PATHWAY	-0.0004	1.22e-04	-3.34	8.83e-04
**Subsample treated with noradrenergic antidepressants, rare + common variants**				
**TRD vs. response**				
**Gene set**	**E**	**SE**	**t**	**p**
GO RESPONSE TO COCAINE	-0.0016	0.0004	-3.73	1.92e-04
GO REGULATION OF INSULIN SECRETION INVOLVED IN CELLULAR RESPONSE TO GLUCOSE STIMULUS	0.0012	0.0004	3.43	6.05e-04
**TRD vs. non-resp vs. response**				
**Gene set**	**E**	**SE**	**t**	**p**
GO RESPONSE TO COCAINE	0.0004	8.91e-05	4.08	5.61e-05

Supplementary Table 9: genes (**A**) and gene sets (**B**) included in the predictive models. The order of predictors corresponds to their relative importance in the model (see also Figure 2).

Supplementary Table 9A

Whole sample	Subset treated with serotonergic antidepressants	Subset treated with noradrenergic antidepressants
HAMP	LYRM1	NFKBIE
ABCD3	CACNA1I	FECH
MTFR1L	SCMH1	CPA6
HPSE	ACSM5	PLAG1
SS18L1	PTPRU	ADGRE5
NBN	REM1	HAMP
BTBD6	ZNF418	NEK8
MRGBP	MTF2	MIR3654
CPA6	RORC	STRBP
BIK	SP7	DBF4B
WWC2-AS2	PRG4	GRID1.AS1
IDH1	BIK	LZTS3
NODAL	ZNF19	NR5A2
SP7	SNRNP25	WDR97
TLCD1	CPSF6	
HSPA9	LPCAT1	
NPBWR2	CEACAM20	
	HPSE	
	LRRN4	
	PDE6G	
	CTNND1	
	HAMP	
	GATS	
	LCE1B	
	FGFBP3	
	PI15	

	RORB.AS1	
	RNASEK-C17orf49	
	ADH1A	
	HIST1H2BK	
	LOC102723824	
	NFKB1	
	AMPD2	
	IQCF5-AS1	
	ARHGAP35	
	MTG2	
	TOM1L1	
	C14orf93	
	PRMT5	
	RHBDF1	
	PLEKHA2	
	ZNF154	
	PTOV1-AS2	
	SIGLEC15	
	FAAP20	
	CCL16	
	PART1	
	TMEM68	
	SPAM1	
	CNOT8	
	PMS2	
	UMPS	
	KIAA1024	
	ZNF366	
	HMGN4	
	LCA5L	
	KRTAP24.1	
	EFCAB2	
	MRPS36	
	DGCR6	
	DUSP23	
	MBIP	
	ZBTB32	
	C1R	
	R3HDM4	
	IQCF5	
	RSBN1L	
	BTBD6	
	GZMM	

	MMP27	
	GUSBP5	
	VDR	
	NODAL	
	SLC17A4	
	EMC4	
	IDH1.AS1	
	TCAF1	
	OGFR	
	ZNF599	
	SMC5	
	WWC2-AS2	
	COL8A1	
	OR5AS1	

Supplementary table 9B

Whole sample	Subset treated with serotonergic antidepressants	Subset treated with noradrenergic antidepressants
GO SEGMENT SPECIFICATION	GO POSITIVE REGULATION OF OXIDOREDUCTASE ACTIVITY	GO COP9 SIGNALOSOME
GO RESPONSE TO IRON ION	GO NEUROPEPTIDE RECEPTOR BINDING	GO SYNAPTIC TRANSMISSION GLUTAMATERGIC
GO PROTEIN IMPORT INTO MITOCHONDRIAL MATRIX	PID CD40 PATHWAY	GO ESTROUS CYCLE
GO POSITIVE REGULATION OF AMINE TRANSPORT	GO LAMIN BINDING	GO RESPIRATORY SYSTEM PROCESS
GO BETA AMYLOID BINDING	GO SIGNAL TRANSDUCTION IN ABSENCE OF LIGAND	GO RESPIRATORY CHAIN
GO INTRA S DNA DAMAGE CHECKPOINT	GO VASCULAR ENDOTHELIAL GROWTH FACTOR RECEPTOR SIGNALING PATHWAY	GO TELOMERASE HOLOENZYME COMPLEX
GO REGULATION OF BONE DEVELOPMENT	GO ORGANIC CATION TRANSPORT	GO AMINOACYL TRNA EDITING ACTIVITY
GO NEGATIVE REGULATION OF DENDRITE DEVELOPMENT	NABA BASEMENT MEMBRANES	GO INTRAMOLECULAR OXIDOREDUCTASE ACTIVITY
GO FIBROBLAST GROWTH FACTOR RECEPTOR BINDING	GO UBIQUINONE METABOLIC PROCESS	GO REGULATION OF HORMONE METABOLIC PROCESS
GO COP9 SIGNALOSOME	REACTOME RAF MAP KINASE CASCADE	GO NEURON NEURON SYNAPTIC TRANSMISSION
GO 3 5 DNA HELICASE ACTIVITY	GO NEGATIVE REGULATION OF DEFENSE RESPONSE	GO REGULATION OF TRANSLATIONAL FIDELITY
	GO REGULATION OF PEPTIDYL SERINE PHOSPHORYLATION OF STAT PROTEIN	GO ISOMERASE ACTIVITY
	GO SEGMENT SPECIFICATION	
	GO RESPONSE TO THYROID HORMONE	
	GO NEGATIVE REGULATION OF DEFENSE RESPONSE TO VIRUS	

	GO POSITIVE REGULATION OF AMINE TRANSPORT	
	GO RESPONSE TO TEMPERATURE STIMULUS	
	GO OUTER MITOCHONDRIAL MEMBRANE PROTEIN COMPLEX	
	GO BASAL LAMINA	
	KEGG ONE CARBON POOL BY FOLATE	
	GO TAU PROTEIN BINDING	
	GO RESPONSE TO ZINC ION	
	GO GLYCERALDEHYDE 3 PHOSPHATE METABOLIC PROCESS	
	GO NEGATIVE REGULATION OF INTRACELLULAR ESTROGEN RECEPTOR SIGNALING PATHWAY	
	GO NUCLEOBASE BIOSYNTHETIC PROCESS	
	REACTOME THE ACTIVATION OF ARYLSULFATASES	
	NABA ECM GLYCOPROTEINS	
	GO EXTRACELLULAR MATRIX	
	REACTOME PROLONGED ERK ACTIVATION EVENTS	
	GO POSITIVE REGULATION OF FILOPODIUM ASSEMBLY	
	GO RESPONSE TO IRON ION	
	GO CILIARY BASAL BODY	
	PID ALK1 PATHWAY	
	GO SULFURIC ESTER HYDROLASE ACTIVITY	
	NABA CORE MATRISOME	

Supplementary Table 10: results referred to non-significant or borderline significant predictive models in the testing sample (GSRD). NA=noradrenergic. 5-HT=serotonergic. AUC=area under the curve, 95% confidence intervals are reported in parenthesis.

Predictors	Phenotype	Sample subset	Results
Pathways, rare variants only	TRD vs. response	NA antidepressants	AUC 0.50 (0.34-0.66)
	Non-response vs. response	Whole testing sample	AUC 0.53 (0.45-0.61)
		5-HT antidepressants	AUC 0.59 (0.48-0.69)
		NA antidepressants	AUC 0.63 (0.46-0.80)
	TRD + non-response vs. response	Whole testing sample	AUC 0.56 (0.48-0.63)
		5-HT antidepressants	AUC 0.58 (0.49-0.67)
		NA antidepressants	AUC 0.44 (0.29-0.58)
Pathways, rare and common variants	TRD vs. response	Whole testing sample	AUC 0.51 (0.44-0.59)
		5-HT antidepressants	AUC 0.53 (0.42-0.64)
		NA antidepressants	AUC 0.58 (0.42-0.74)

	Non-response vs. response	Whole testing sample	AUC 0.56 (0.48-0.64)
		5-HT antidepressants	AUC 0.61 (0.51-0.72)
		NA antidepressants	AUC 0.60 (0.43-0.77)
	TRD + non-response vs. response	Whole testing sample	AUC 0.48 (0.41-0.55)
		5-HT antidepressants	AUC 0.52 (0.43-0.61)
		NA antidepressants	AUC 0.61 (0.46-0.76)
Genes, rare and common variants	TRD vs. response	NA antidepressants	AUC 0.60 (0.45-0.76)
	Non-response vs. response	Whole testing sample	AUC 0.59 (0.51-0.67)
		5-HT antidepressants	AUC 0.64 (0.537-0.74)
		NA antidepressants	AUC 0.65 (0.48-0.82)
	TRD + non-response vs. response	Whole testing sample	AUC 0.59 (0.51-0.65)
		5-HT antidepressants	AUC 0.52 (0.42-0.61)
		NA antidepressants	AUC 0.57 (0.42-0.72)

Supplementary Table 11: clinical-demographic characteristics of patients included from GENDEP and STAR*D studies. Only patients having both the exome and genome-wide arrays were considered. QIDS-CR=Quick Inventory of Depressive Symptomatology Clinician-Rated.

**Variable**	**GENDEP (n=756)**	**STAR*D (n=959)**
Age	42.18±11.52	42.79±13.58
Gender F/M	470/286	553/406
Baseline depression severity	28.85±6.75	16.28±3.17 (QIDS-CR)
Treatment	escitalopram (57%), nortriptyline (43%)	Level 1: citalopram<br>Level 2: bupropion (15%), citalopram+bupropion (19%), citalopram + buspirone (22%), citalopram + cognitive therapy (5.5%), cognitive therapy (3.2%), sertraline (17%), venlafaxine (18.3%)
Number of previous depressive episodes	1.73±0.68	4.69±5.53
Duration of the current depressive episode	21.17± 17.29 (weeks)	1.21±1.60 (years)
Chronic depression yes/no	56/700 (>= 1 year)	184/732 (>= 2 years)
Suicidality yes/no	159/596	240/719
Phenotype distribution	TRD=103; non-responders=435; responder=218	TRD=243; non-responders=145; responders=571

Supplementary Table 12: application of the significant predictive models developed in GSRD training set in STAR*D and GENDEP. Extreme genetic percentiles were considered ≤ 20 or ≥ 80 percentiles. *Significant models.

**Pathway scores as predictors – rare variants**			
**Whole STAR*D sample**			
**Genetic predictors only**	**Genetic predictors + clinical risk score**	**Extreme genetic percentiles, genetic predictors only**	**Extreme genetic percentiles, genetic predictors + clinical score**
AUC 0.53 (0.49-0.58)	AUC 0.54 (0.49-0.58)	AUC 0.55 (0.48-0.62)	AUC 0.52 (0.45-0.60)
**Whole GENDEP sample**			
**Genetic predictors only**	**Genetic predictors + clinical risk score**	**Extreme genetic percentiles, genetic predictors only**	**Extreme genetic percentiles, genetic predictors + clinical score**
AUC 0.54 (0.47-0.60)	AUC 0.60 (0.54-0.65)*<br>Sens=0.64; spec=0.52; PPV=0.39; NPV=0.75	AUC 0.50 (0.39-0.61)	AUC 0.59 (0.47-0.70)
**STAR*D sample – patients treated with 5-HT antidepressants**			

**Genetic predictors only**	**Genetic predictors + clinical risk score**	**Extreme genetic percentiles, genetic predictors only**	**Extreme genetic percentiles*, genetic predictors + clinical score**
AUC 0.52 (0.46-0.59)	AUC 0.54 (0.48-0.60)	AUC 0.59 (0.48-0.69)	AUC 0.61 (0.51-0.71)*<br>Sens=0.35; spec=0.85; PPV=0.29; NPV=0.88
**GENDEP sample – patients treated with 5-HT antidepressants**			
**Genetic predictors only**	**Genetic predictors + clinical risk score**	**Extreme genetic percentiles, genetic predictors only**	**Extreme genetic percentiles, genetic predictors + clinical score**
AUC 0.55 (0.46-0.59)	AUC 0.50 (0.40-0.59)	AUC 0.58 (0.38-0.77)	AUC 0.52 (0.34-0.69)
**Gene scores as predictors – rare and common variants**			
**Whole STAR*D sample**			
**Genetic predictors only**	**Genetic predictors + clinical risk score**	**Extreme genetic percentiles, genetic predictors only**	**Extreme genetic percentiles, genetic predictors + clinical score**
AUC 0.51 (0.46-0.55)	AUC 0.55 (0.51-0.59)*<br>Sens=0.38; spec=0.72; PPV=0.36; NPV=0.73	AUC 0.52 (0.45-0.59)	AUC 0.50 (0.42-0.56)
**Whole GENDEP sample**			
**Genetic predictors only**	**Genetic predictors + clinical risk score**	**Extreme genetic percentiles, genetic predictors only**	**Extreme genetic percentiles, genetic predictors + clinical score**
AUC 0.50 (0.42-0.56)	AUC 0.54 (0.48-0.61)	AUC 0.53 (0.42-0.64)	AUC 0.56 (0.45-0.66)
**STAR*D sample – patients treated with 5-HT antidepressants**			
**Genetic predictors only**	**Genetic predictors + clinical risk score**	**Extreme genetic percentiles, genetic predictors only**	**Extreme genetic percentiles, genetic predictors + clinical score**
AUC 0.55 (0.49-0.62)	AUC 0.55 (0.48-0.61)	AUC 0.55 (0.44-0.65)	AUC 0.53 (0.43-0.64)
**GENDEP sample – patients treated with 5-HT antidepressants**			
AUC 0.58 (0.49-0-68)	AUC 0.62 (0.53-0.72)*<br>Sens=0.60; spec=0.67; PPV=0.36; NPV=0.84	AUC 0.58 (0.40-0.75)	AUC 0.57 (0.40-0.0.75)

Supplementary Figure 1: beta distributions used to estimate frequency-based weights for rare (**A**) and common variants (**B**).

A dbeta(alt_allele_freq,1,25) **B** dbeta(alt_allele_freq,0.5,0.5)

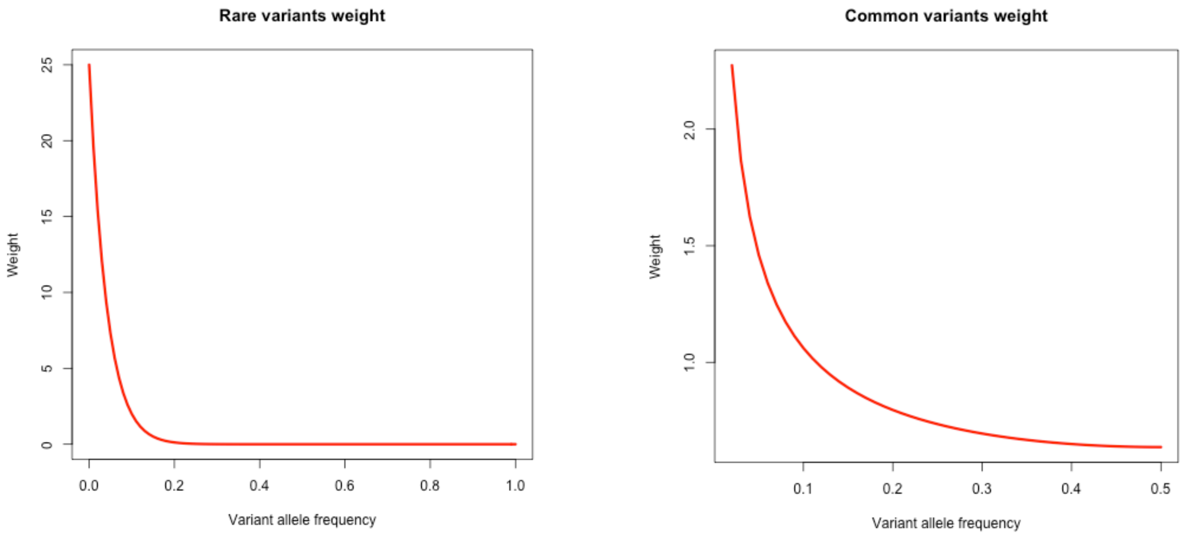

Supplementary Figure 2: Flowchart of the number of patients excluded during quality control.

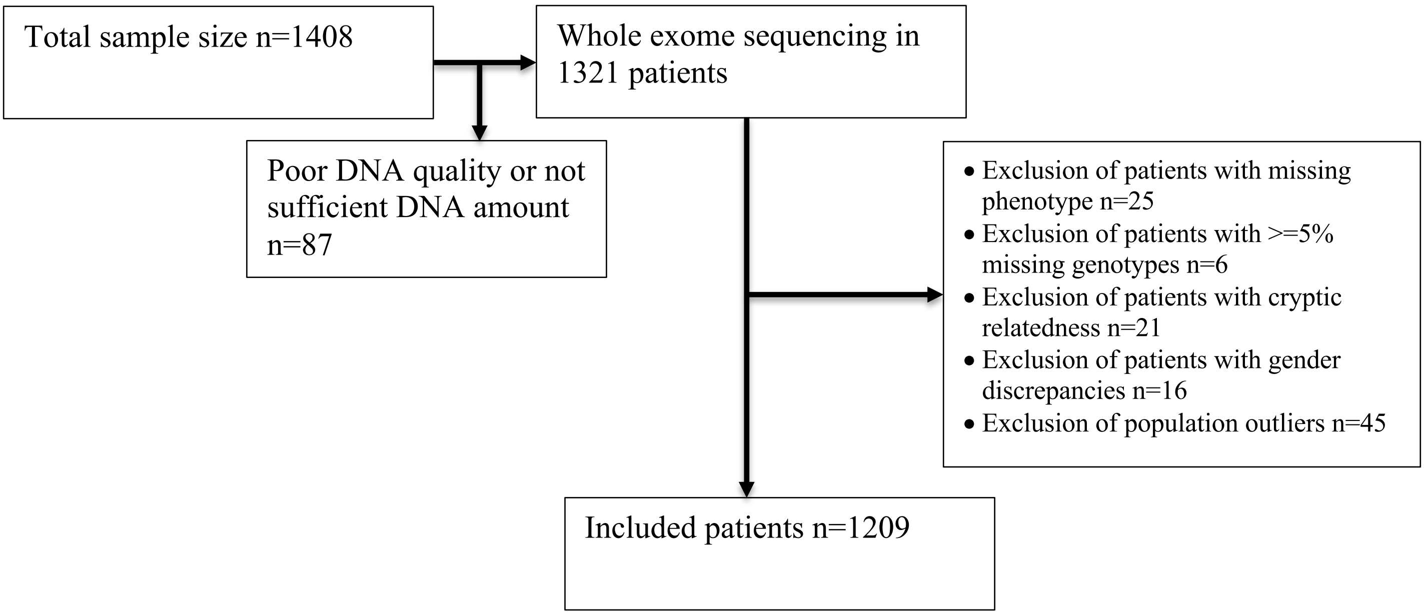

Supplementary Figure 3: concordance between rare variants from exome sequencing and rare variants (genotyped or imputed) from genome-wide genotyping. The total number of variants available for these comparisons was 161,130 (120,632 imputed variants and 40,497 genotyped variants).

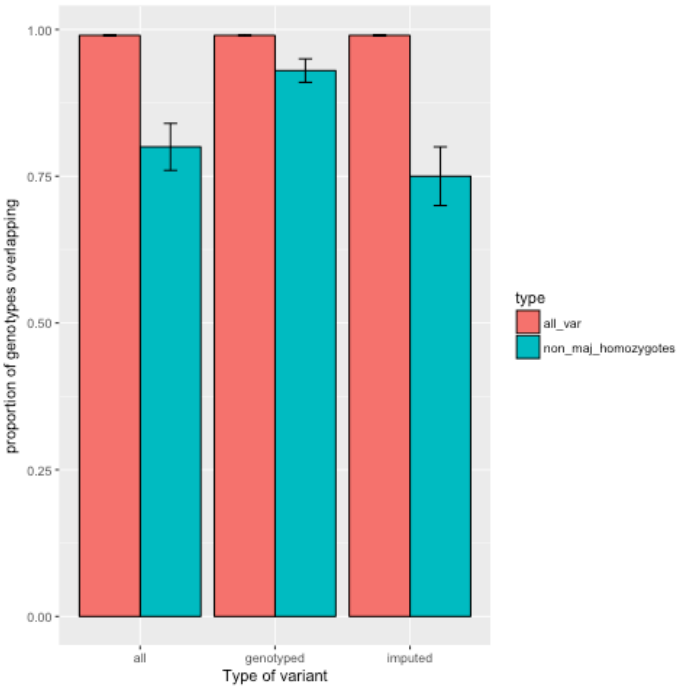

Supplementary Figure 4: exome-wide distribution of the score (re-scaled between 20 and 100) using the described different weighting approaches. Subjects with scores outside 5 SD from the mean (red lines) were excluded from the subsequent analyses. SO=sequence ontology project.

Eigen weights **CADD weights**

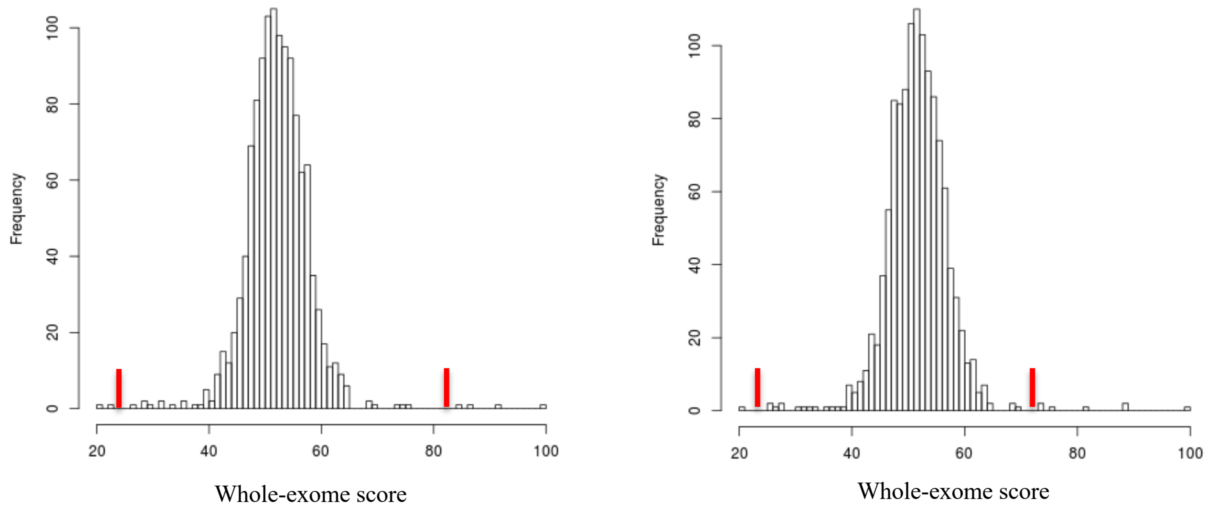

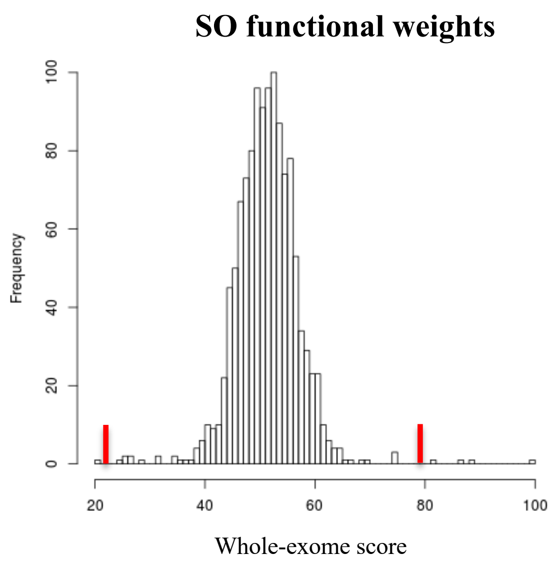

Supplementary Figure 5: correlation (Pearson's correlation coefficient) among gene scores obtained using different functional weighting.

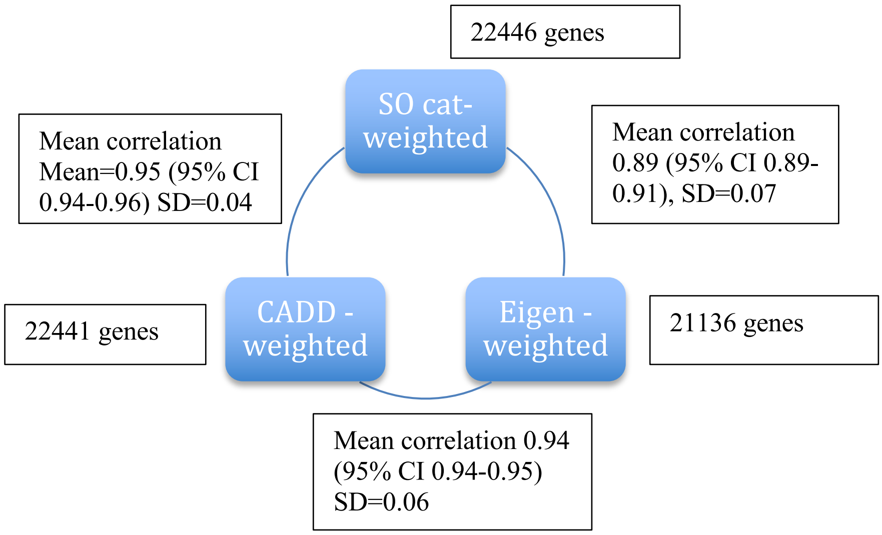

Supplementary Figure 6: contribution of predictors included in the developed models (scaled between 0 and 100). For models including more than 20 predictors, the first 20 are shown.

Genetic predictors only	Genetic and clinical predictors

Whole testing sample, rare variant pathway-based scores

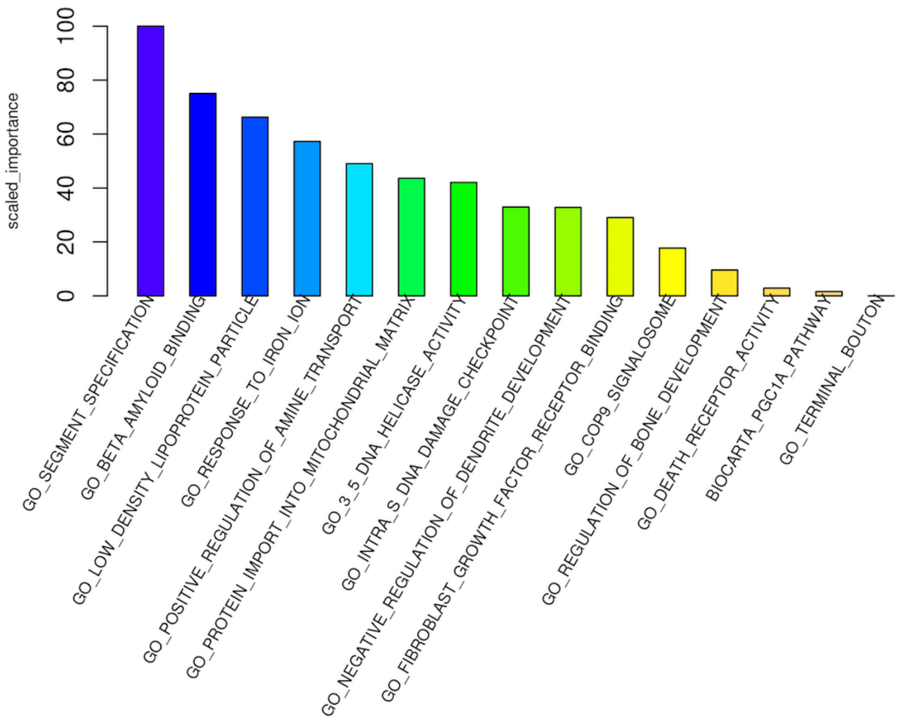

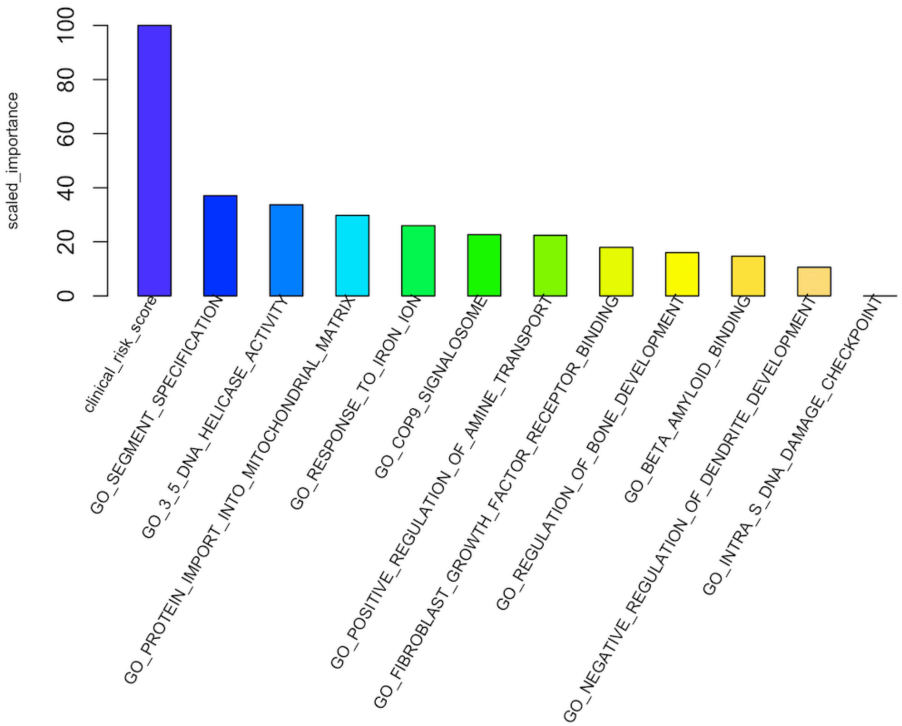

Genetic predictors only	Genetic and clinical predictors

5-HT antidepressant treated testing sample, rare variant pathway-based scores

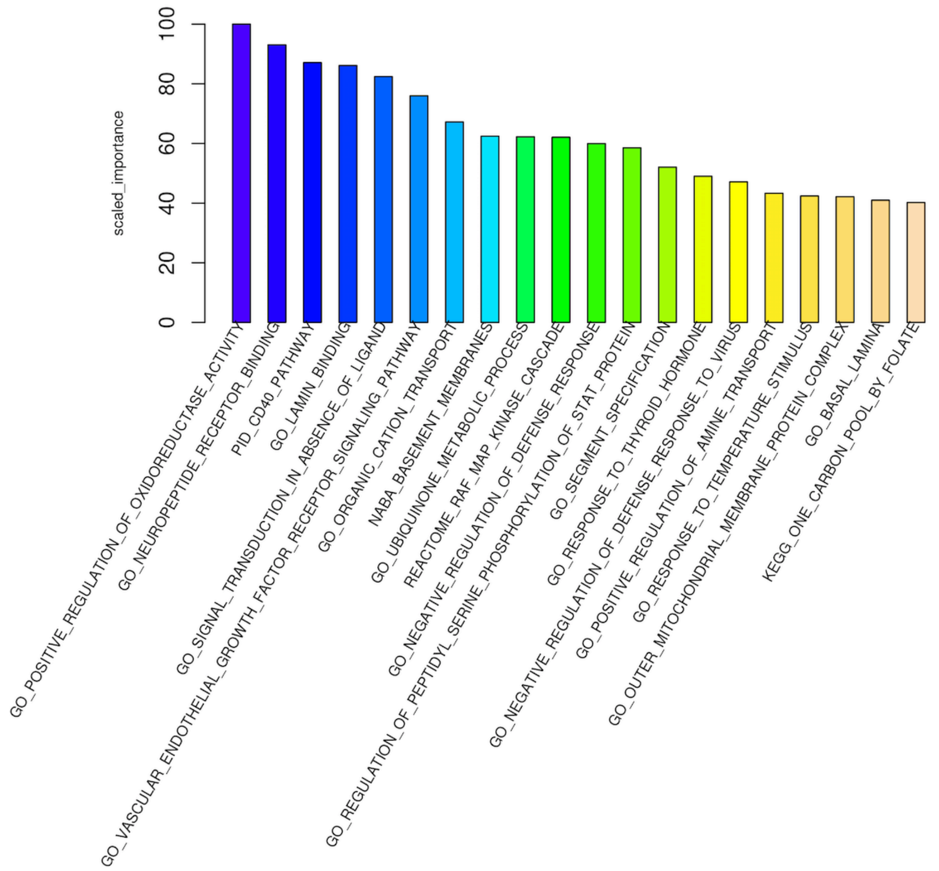

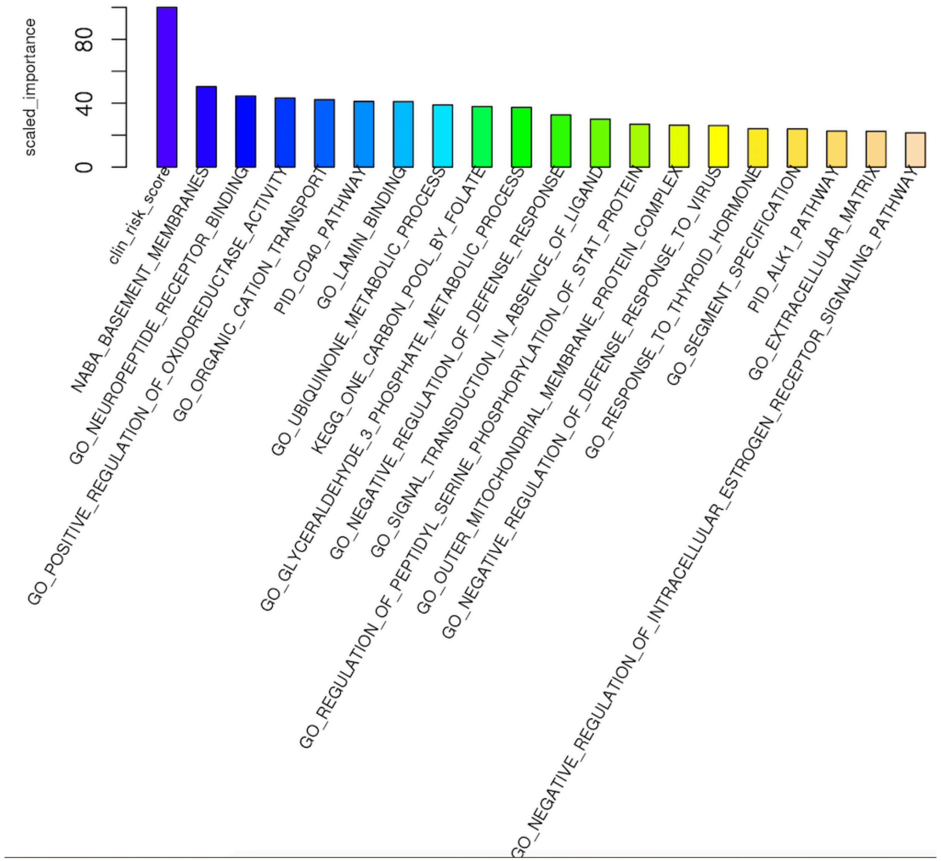

Genetic predictors only | **Genetic and clinical predictors**

Whole testing sample, rare-common variant gene-based scores

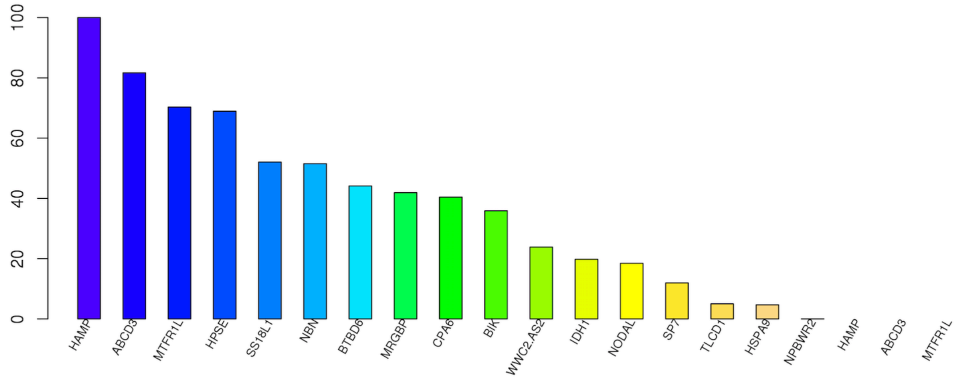

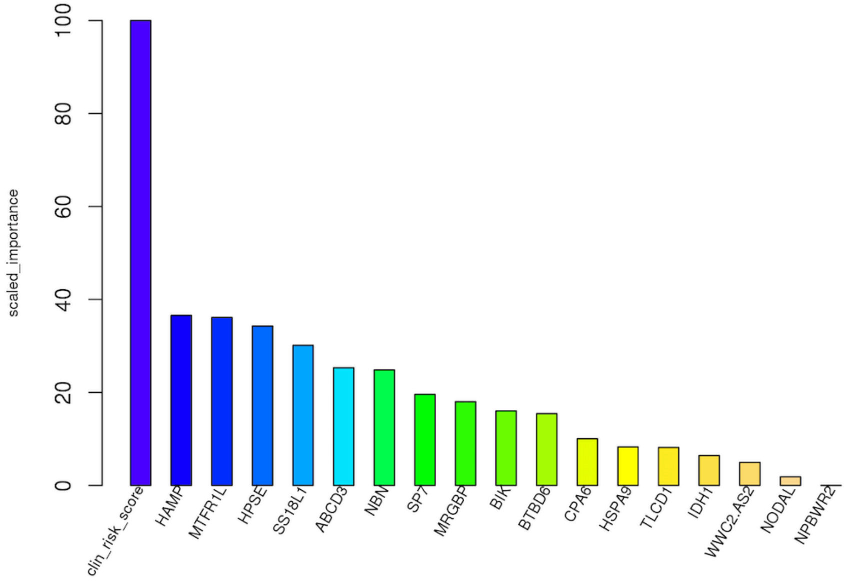

5-HT antidepressant treated testing sample, rare-common variant gene-based scores

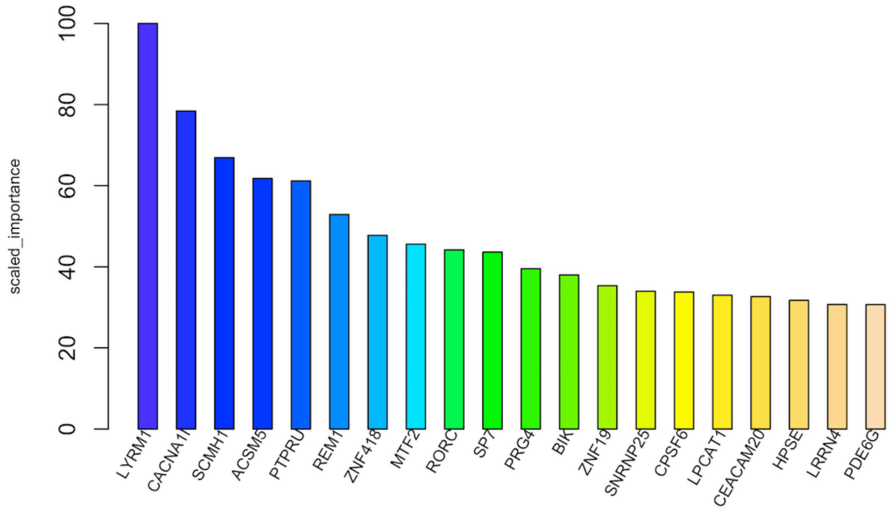

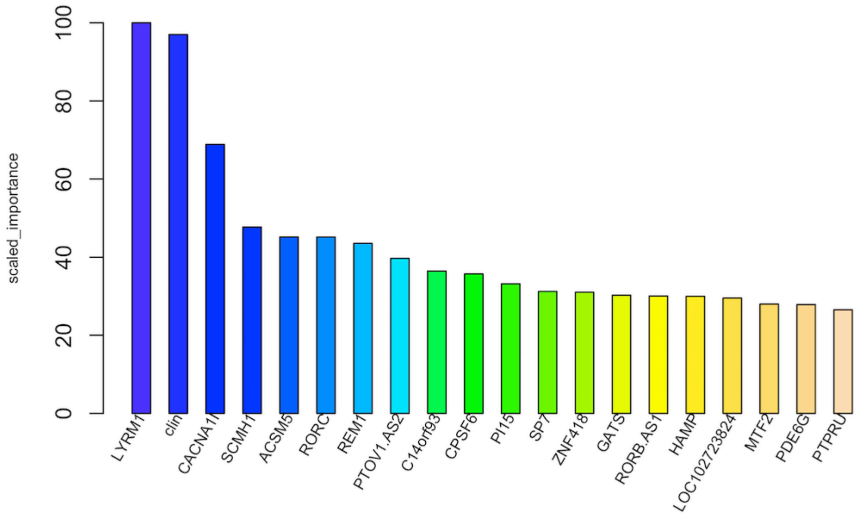

Supplementary Figure 7: comparison of the distribution of rare variants between the GSRD sample (exome sequence data) and GENDEP (genome-wide and exome arrays after imputation).

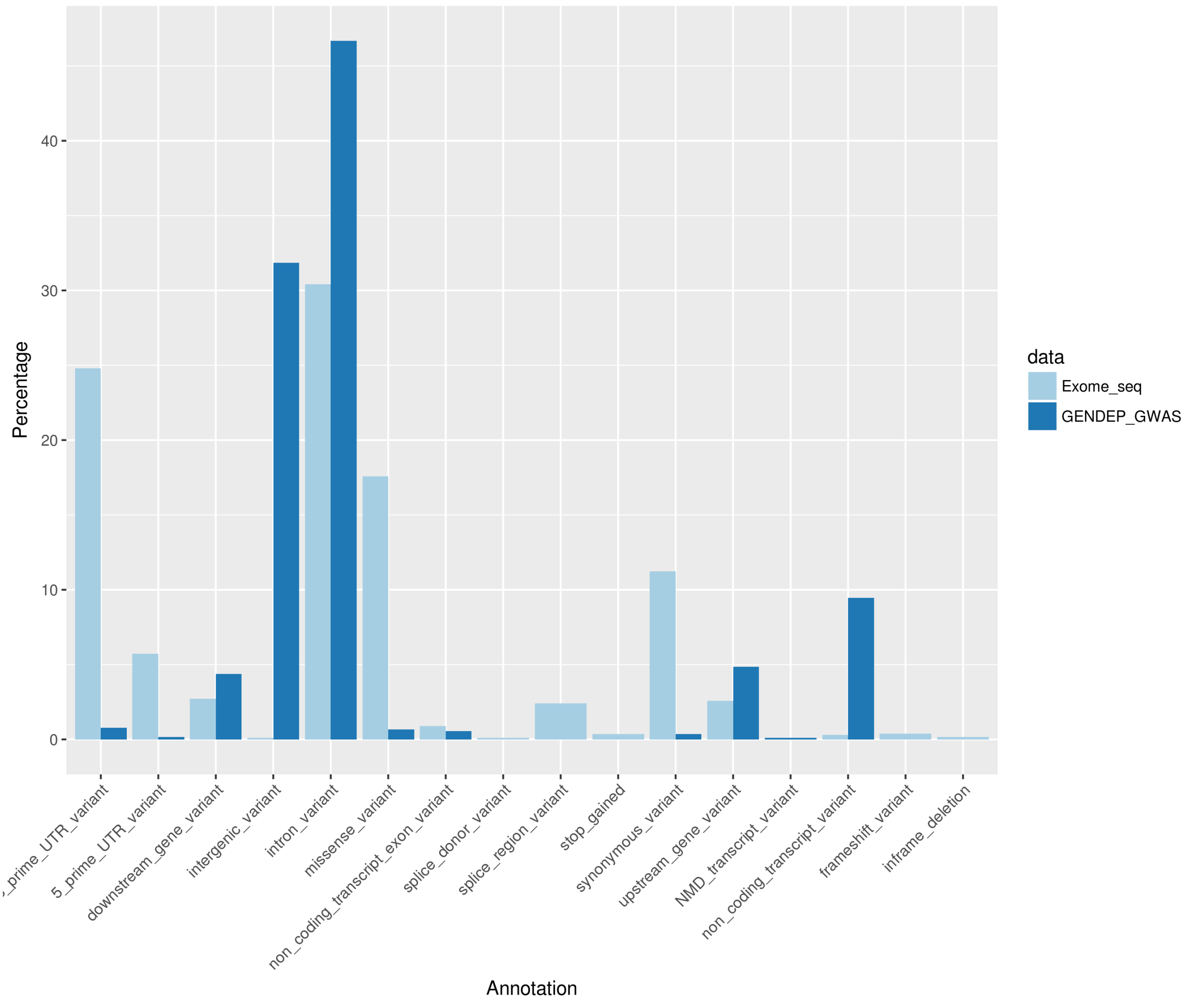

References

1. Babraham Bionformatics. FastQC: A quality control tool for high throughput sequence data [Internet]. [cited 2019 Jun 18]. Available from: http://www.bioinformatics.babraham.ac.uk/projects/fastqc/
2. FASTX-Toolkit. FASTQ/A Trimmer [Internet]. [cited 2019 Jun 18]. Available from: http://hannonlab.cshl.edu/fastx_toolkit/index.html
3. Kim D, Pertea G, Trapnell C, Pimentel H, Kelley R, Salzberg SL. TopHat2: accurate alignment of transcriptomes in the presence of insertions, deletions and gene fusions. Genome Biol. 2013;14(4):R36.
4. Garrison E, Sibley T, Lee D, Marks P, Spies N, Randall J, et al. Freebayes, a haplotype-based variant detector [Internet]. [cited 2019 Jun 18]. Available from: https://github.com/ekg/freebayes
5. Zhang S, Wang B, Wan L, Li LM. Estimating Phred scores of Illumina base calls by logistic regression and sparse modeling. BMC Bioinformatics. 2017;18(1):335.
6. Wang Q, Shashikant CS, Jensen M, Altman NS, Girirajan S. Novel metrics to measure coverage in whole exome sequencing datasets reveal local and global non-uniformity. Sci Rep. 2017;7(1):885.

7. Fang H, Wu Y, Narzisi G, et al. Reducing INDEL calling errors in whole genome and exome sequencing data. Genome Med. 2014;6(10):89.
8. Hutter S, Piro RM, Waszak SM, et al. No correlation between NF1 mutation position and risk of optic pathway glioma in 77 unrelated NF1 patients. Hum Genet. 2016;135(5):469–75.
9. Anderson CA, Pettersson FH, Clarke GM, Cardon LR, Morris AP, Zondervan KT. Data quality control in genetic case-control association studies. Nat Protoc. 2010;5(9):1564–73.
10. Zhang Y, Shen X, Pan W. Adjusting for population stratification in a fine scale with principal components and sequencing data. Genet Epidemiol. 2013;37(8):787–801.
11. Fabbri C, Kasper S, Kautzky A, et al. Genome-wide association study of treatment-resistance in depression and meta-analysis of three independent samples. Br J Psychiatry. 2019;214(1):36–41.
12. McCarthy S, Das S, Kretzschmar W, et al. A reference panel of 64,976 haplotypes for genotype imputation. Nat Genet. 2016;48(10):1279–83.
13. Das S, Forer L, Schönherr S, et al. Next-generation genotype imputation service and methods. Nat Genet. 2016;48(10):1284–7.
14. Kircher M, Witten DM, Jain P, O'Roak BJ, Cooper GM, Shendure J. A general framework for estimating the relative pathogenicity of human genetic variants. Nat Genet. 2014;46(3):310–5.
15. Ionita-Laza I, McCallum K, Xu B, Buxbaum JD. A spectral approach integrating functional genomic annotations for coding and noncoding variants. Nat Genet. 2016;48(2):214–20.
16. Curtis D. Construction of an Exome-Wide Risk Score for Schizophrenia Based on a Weighted Burden Test. Ann Hum Genet. 2018;82(1):11–22.
17. Liberzon A, Birger C, Thorvaldsdóttir H, Ghandi M, Mesirov JP, Tamayo P. The Molecular Signatures Database (MSigDB) hallmark gene set collection. Cell Syst. 2015;1(6):417–25.
18. Pirooznia M, Wang T, Avramopoulos D, et al. SynaptomeDB: an ontology-based knowledgebase for synaptic genes. Bioinformatics. 2012;28(6):897–9.
19. Wray NR, Ripke S, Mattheisen M, et al. Genome-wide association analyses identify 44 risk variants and refine the genetic architecture of major depression. Nat Genet. 2018;50(5):668–81.
20. Pardiñas AF, Holmans P, Pocklington AJ, et al. Common schizophrenia alleles are enriched in mutation-intolerant genes and in regions under strong background selection. Nat Genet. 2018;50(3):381–9.
21. Purcell SM, Moran JL, Fromer M, et al. A polygenic burden of rare disruptive mutations in schizophrenia. Nature. 2014;506(7487):185–90.
22. Stahl EA, Breen G, Forstner AJ, et al. Genome-wide association study identifies 30 loci associated with bipolar disorder. Nat Genet. 2019;51(5):793–803.
23. Wang X, Zhang D, Tzeng J-Y. Pathway-guided identification of gene-gene interactions. Ann Hum Genet. 2014;78(6):478–91.
24. Rush AJ, Fava M, Wisniewski SR, et al. Sequenced treatment alternatives to relieve depression (STAR*D): rationale and design. Control Clin Trials. 2004;25(1):119–42.
25. Uher R, Perroud N, Ng MYM, et al. Genome-wide pharmacogenetics of antidepressant response in the GENDEP project. Am J Psychiatry. 2010;167(5):555–64.
26. Iniesta R, Malki K, Maier W, et al. Combining clinical variables to optimize prediction of antidepressant treatment outcomes. J Psychiatr Res. 2016;78:94–102.
27. Garriock HA, Kraft JB, Shyn SI, et al. A Genomewide Association Study of Citalopram Response in Major Depressive Disorder. Biological Psychiatry. 2010;67(2):133–8.
28. Patterson N, Price AL, Reich D. Population structure and eigenanalysis. PLoS Genet. 2006;2(12):e190.
29. Ionita-Laza I, Lee S, Makarov V, Buxbaum JD, Lin X. Sequence kernel association tests for the combined effect of rare and common variants. Am J Hum Genet. 2013;92(6):841–53.
30. Khan A, Dager SR, Cohen S, Avery DH, Scherzo B, Dunner DL. Chronicity of depressive episode in relation to antidepressant-placebo response. Neuropsychopharmacology. 1991;4(2):125–30.

31. Uher R, Perlis RH, Henigsberg N, et al. Depression symptom dimensions as predictors of antidepressant treatment outcome: replicable evidence for interest-activity symptoms. Psychol Med. 2012;42(5):967–80.
